# Screening Novel Vaccine Candidates for *Leishmania Donovani* by Combining Differential Proteomics and Immunoinformatics Analysis

**DOI:** 10.3389/fimmu.2022.902066

**Published:** 2022-06-23

**Authors:** Jianhui Zhang, Jiao Li, Kaifeng Hu, Qi Zhou, Xiaoxiao Chen, Jinlei He, Shuangshuang Yin, Yangjian Chi, Xuechun Liao, Yuying Xiao, Hanxiao Qin, Zhiwan Zheng, Jianping Chen

**Affiliations:** ^1^ Department of Pathogenic Biology, West China School of Basic Medical Sciences and Forensic Medicine, Sichuan University, Chengdu, China; ^2^ Department of Clinical Medicine, Guangzhou University of Chinese Medicine, Guangzhou, China; ^3^ Department of Obstetrics and Gynecology, The First Affiliated Hospital of Guangzhou University of Chinese Medicine, Guangzhou, China; ^4^ Department of Urinary Surgery, Jianou Municipal Hospital of Fujian Province, Jianou, China; ^5^ Animal Disease Prevention and Food Safety Key Laboratory of Sichuan Province, Sichuan University, Chengdu, China

**Keywords:** *Leishmania donovani*, vaccine, immunoinformatics, differentially expressed protein, epitope prediction

## Abstract

Visceral leishmaniasis (VL), also known as kala-azar, is the most dangerous form of leishmaniasis. Currently no effective vaccine is available for clinical use. Since the pathogenicity of different *Leishmania* strains is inconsistent, the differentially expressed proteins in *Leishmania* strains may play an important role as virulence factors in pathogenesis. Therefore, effective vaccine candidate targets may exist in the differentially expressed proteins. In this study, we used differential proteomics analysis to find the differentially expressed proteins in two *Leishmania donovani* strains, and combined with immunoinformatics analysis to find new vaccine candidates. The differentially expressed proteins from *L*. DD8 (low virulent) and *L*. 9044 (virulent) strains were analyzed by LC-MS/MS, and preliminarily screened by antigenicity, allergenicity and homology evaluation. The binding peptides of MHC II, IFN-γ and MHC I from differentially expressed proteins were then predicted and calculated for the second screening. IFN-γ/IL-10 ratios and conserved domain prediction were performed to choose more desirable differentially expressed proteins. Finally, the 3D structures of three vaccine candidate proteins were produced and submitted for molecular dynamics simulation and molecular docking interaction with TLR4/MD2. The results showed that 396 differentially expressed proteins were identified by LC-MS/MS, and 155 differentially expressed proteins were selected through antigenicity, allergenicity and homology evaluation. Finally, 16 proteins whose percentages of MHC II, IFN-γ and MHC I binding peptides were greater than those of control groups (TSA, LmSTI1, LeIF, Leish-111f) were considered to be suitable vaccine candidates. Among the 16 candidates, amino acid permease, amastin-like protein and the hypothetical protein (XP_003865405.1) simultaneously had the large ratios of IFN-γ/IL-10 and high percentages of MHC II, IFN-γ and MHC I, which should be focused on. In conclusion, our comprehensive work provided a methodological basis to screen new vaccine candidates for a better intervention against VL and associated diseases.

## Introduction

Visceral leishmaniasis (VL), the most dangerous form of leishmaniasis, is mainly characterized by irregular fever, hepatosplenomegaly, and anemia, which is fatal without proper treatment. *Leishmania donovani* (*L. donovani*) and *Leishmania infantum* (*L. infantum*) are causative agents of VL and transmit to humans and vertebrates with sand flies. At present, it was reported by World Health Organization (WHO) that there were at least 50,000 to 90,000 new cases of VL occurred annually globally and more than 90% of new cases in 2020 were found in Brazil, China, Ethiopia, India, and so on (1). Thus, leishmaniasis is the second vital vector-borne protozoal disease after malaria and more measures should be taken to control it (2). Drugs available for VL, are associated with toxicity, high cost, and drug resistance (3–5). Most individuals recovering from VL develop immune protection against *leishmania* and become resistant to later clinical reinfection for a long time, indicating that control of the disease by vaccine is a viable approach (6, 7). However, no licensed vaccines are currently available for clinical use to prevent the infection by *Leishmania* species. Hence, there is an urgent need to develop an effective vaccine.

Proteomics was applied to evaluate protein expression in *leishmania* and offered the possibility of virulence, vaccine candidate, diagnostic markers, and immunotherapeutic target recognition (8). The proteomic studies were performed in *Leishmania* to evaluate the protein expression of different stages and species (9–11). Proteins contributing to the infectivity should be vital because they are considered as potential vaccine candidates and drug targets against the disease (12). The differentially expressed proteins in *leishmania* from different stages or species were identified to gain insight into the mechanisms supporting survival, which led to the identification of novel vaccine candidates and therapeutic targets (8, 13, 14). For example, Fakhry et al. found that more than 62 differentially expressed proteins from Leishmania different stages were detected. Among them, two highly expressed proteins (isocitrate dehydrogenase and triosepho-sphate isomerase) in amastigote might act as virulence factors because they respectively involved with Glucose metabolism pathways and the processes of NADPH and a-ketoglutarate production to support parasitic intracellular survival, which permitted the possibility of new vaccine and drug development (13).

Reverse vaccinology is the technology employed for new antigen identifications (15, 16). It analyzes amino acid sequences from open reading frames computationally. Proteins homologous to humans are abandoned, and the rest are predicted for immunogenicity (17). With the rapid development of bioinformatics, a series of feasible methods have been used in reverse vaccinology. It is prevalent that amino acid sequences are subjected to the predictions of helper T lymphocyte, cytotoxic T lymphocyte, and B cell epitopes, which saves lots of time and cost for vaccine research (18). Through the prediction of the epitope, many studies usually combined plenty of epitopes from different target proteins to construct multi-epitope vaccines, such as COVID-19, *Leishmania*, and *Schistosoma* (2, 19, 20). However, few studies have used bioinformatics to discover novel proteins of vaccine potential.

The greater richness the T cell epitopes contained within a protein, the more possibility it will induce an immune response (21). In our present research, based on differential proteomics and bioinformatics, reverse vaccinology was conducted for new antigens with rich T cell epitopes from *Leishmania*. The differentially expressed proteins of *L*. 9044 (virulent) and *L*. DD8 (low virulent) strains of *L. donovani* were analyzed to find new desirable vaccine candidates. These proteins were examined to remove the proteins with antigenic index< 0.5, high allergenicity, and the similarity to humans and mice. The remaining differentially expressed proteins were subjected to predict MHC II, IFN-γ, and MHC I epitopes and calculated the percentages of MHC II, IFN-γ, and MHC I binding peptides. LeIF (the *Leishmania* elongation initiation factor), TSA (Thiol-specific antioxidant), LmSTI1 (*Leishmania major* homologue to eukaryotic stress-inducible protein), and Leish-111f composed of LeIF, TSA, and LmSTI-1 were employed as control groups (22–25). Differentially expressed proteins with higher percentages of MHC II, IFN-γ, and MHC I binding peptides than those of control groups were selected as the proteins suitable for vaccine candidate. In addition, the ratios of IFN-γ/IL-10 and conserved domains were evaluated *in silico*. According to those results, vaccine candidates were selected for further analyses including tertiary structure, molecular docking, and molecular dynamic simulation.

## Materials and Methods

### Parasites and Animals

Three eight-week-old BALB/c mice were prepared from Dassy experimental animals Co., Ltd (Chengdu, China). *L.* DD8 (MHOM/IN/80/DD8) and *L.* 9044 (MHOM/CN/90/9044) were cultured in M199 (HyClone, USA) medium with 10% fetal bovine serum (FBS, Gibco, USA), antibiotics (100 U/ml penicillin and 100 μg/ml streptomycin) at 22°C. For experiments, all BALB/c mice were sacrificed by intraperitoneal injections of pentobarbital sodium and cervical dislocation.

### Parasitic Infection *In Vitro*

To confirm the difference between *L*. DD8 and *L*. 9044 strains in resistance to immune clearance, Three BALB/c mice were sacrificed for peritoneal macrophages following the description above. Ice-cold RPMI 1640 medium was injected into the peritoneal cavity for 15 min to gain the peritoneal macrophages. The cells were then cultured with RPMI 1640 containing 10% FBS in 12-well plates at 37°C in 5% CO_2_. After 8 hours, the cells were washed with PBS to remove non-adherent cells and the adherent cells were prepared for infected experiments. To determine the virulent differences between *L.* 9044 and *L.* DD8, promastigotes of *L*. 9044 and *L*. DD8 were respectively co-cultured with peritoneal macrophages at a ratio of 10:1 (promastigotes to cells), and the parasites which not phagocytized by peritoneal macrophages were washed away with PBS at 6 h. Subsequently, the cells were stained by Wright’s staining for the evaluation of parasitic burdens at the 6, 12, 18, and 24 h. The number of parasites per 100 cells was counted under light microscopes using 1000 power magnification.

### Differentially Expressed Proteins Generation and Analysis

The protein data of *L. donovani* 9044 and DD8 strains was generated by using liquid chromatography-mass spectrometry/mass spectrometry (LC-MS/MS) in our previous studies (26). Proteomic data was not employed for immunoinformatics analysis in previous studies and it can be retrieved from ProteomeXchange Consortium *via* the accession number PXD017089. In brief, *L.* 9044 and *L.* DD8 were cultured and collected to incubate in lysis buffer (8 M urea and 1% protease inhibitor cocktail) at 4°C for 3 min. Lysis samples were sonicated on ice three times using a high-intensity ultrasonic processor (Scientz, Ningbo, China) and the supernatant was collected by centrifugation for an insolution reduction, alkylation, and digestion approach. The processed samples were dissolved in 1.0% (v/v) formic acid, and then subjected to liquid chromatography-mass spectrometry/mass spectrometry (LC-MS/MS) analysis using a QExactiveTM Plus Orbitrap mass spectrometer (Thermo Fisher Scientific) coupled online to the EASY-nLC 1000 UPLC system. Perseus software v.1.6.15.0 was employed to determine differentially expressed proteins (Fold change ≥ 3, q-value < 0.01) between *L.* 9044 and *L.* DD8.

### Prediction of Antigenicity, Allergenicity, and Human and Mice Homologous Proteins

Vaxijen (http://www.ddgpharmfac.net/vaxijen/VaxiJen/VaxiJen.html) is usually applied to evaluate the antigenicity of protein and has an accuracy from 70 to 89% (27). The differentially expressed proteins were subjected to Vaxijen for the prediction of antigenicity and the differentially expressed proteins having a score of > the threshold value 0.5 were chosen. Allergenic proteins are involved in hypersensitive reaction and the proteins similar to hosts may result in autoimmune responses, which will be harmful to organisms. The proteins that are non-allergic and heterologous to hosts are appropriate for vaccine development. The analyses of allergenicity were performed by the AlgPred server (http://www.imtech.res.in/raghava/algpred) with a hybrid approach (SVMc+IgE epitope+ARPs BLAST+MAST). Identifying the similarity of humans and mice proteins was done with the Blastp alignments. The similar proteins (sequence identify >30% and expect value (E-value) <1e-5 cutoff in Blastp alignments) were flited out. Then, the results selected from this measure were be predicted for their MHC II, MHC I, and IFN-γ epitopes.

### MHC II Epitope Prediction

The predictions of MHC II epitope from differentially expressed proteins were performed using Immune Epitope Database server (IEDB; http://tools.iedb.org/mhcii/, with 85% accuracy) based on a recommended method that combined NN-align, SMM-align, CombLib, Sturniolo, and NetMHCIIpan methods. Fifteen mer length epitopes were predicted for 27 alleles of human leukocyte antigen II (HLA II) and the epitopes with percentile rank < 10 were considered as binding peptides. The average percentage of 27 alleles binding peptides was calculated as follow:


$$\text{ the average percentage of }27\text{ allele binding peptides}=\frac{\text{the sum of binding peptides from }27\text{ alleles}}{\text{No}.\text{ of total peptides}\times 27}$$

LeIF, TSA, LmSTI1, and Leish-111f as control groups were subjected to IEDB to analyze their average percentage of 27 allele binding peptides. The differentially expressed proteins, having higher average percentages of MHC II binding peptides than those of controls, were considered for further analyses.

### IFN-γ Inducing Epitopes Prediction

The identification of IFN-γ inducing epitopes was performed using the IFN-γ epitope server (http://crdd.osdd.net/raghava/ifnepitope/, with 81.39% accuracy) (28). The MHC II binding peptides from differentially expressed proteins were submitted to this server for the prediction of IFN-γ epitope based on the support vector machine (SVM) hybrid approach. The positive epitopes were considered as IFN-γ inducing peptides and the percentage of IFN-γ inducing peptides was calculated as follow:


$$\text{the percentage of IFN}-\text{γ inducing peptides}=\frac{\text{No}.\text{ of IFN}-\text{γ inducing epitopes}}{\text{No}.\text{ of total MHC II binding peptides}}$$

LmSTI1, LeIF, TSA, and Leish-111f were employed as control groups for IFN-γ inducing peptide predictions. The differentially expressed proteins, having higher percentages of IFN-γ inducing peptides than those of controls, were chosen for further analyses.

### MHC I Epitope Prediction

NetMHC-4.0 server (https://services.healthtech.dtu.dk/service.php?NetM HC-4.0, with 86% accuracy) was used to predict MHC I epitopes from differentially expressed proteins. This server was founded on artificial neural networks (ANN) with a sequence alignment method and predicted 9 mer length epitopes of human leukocyte antigen I (HLA I). Three supertypes (A2, A3, and B7) covering 90% of the global population were predicted and the epitopes containing a half-maximal inhibitory concentration (IC_50_) < 500 nm were recognized as binding peptides (29). The average percentage of three MHC I supertype binding peptides was calculated as follows:


$$\text{the average percentage of MHC I binding peptides}=\frac{\text{the sum of binding peptides from three supertypes}}{\text{No}.\text{ of total peptides}\times 3}$$

LeIF, LmSTI1, TSA, and Leish-111f were employed as control groups for MHC I binding peptide predictions. The differentially expressed proteins, having higher percentages of MHC I binding peptides than those of controls, were selected for further analyses.

### The Evaluation of the Ratio of IFN-γ to IL-10 and Conserved Domain Analysis

After epitope prediction, the differentially expressed proteins whose percentages of MHC II, MHC I, and IFN-γ epitope were all more than those of control groups (LmSTI1, LeIF, TSA, and Leish-111f) were taken to immune response simulations for the evaluation of the ratio of IFN-γ to IL-10. The dynamics simulation of immune response *in silico* was conducted using the C-ImmSim server (https://kraken.iac.rm.cnr.it/C-IMMSIM/). To stimulate both humoral and cellular responses against selected differentially expressed proteins, C-ImmSim utilizes a position-specific scoring matrix (PSSM) for epitope recognition and machine learning techniques for the simulation of immune interactions (30). The simulation of this server involves three distinct anatomical regions: bone marrow, the thymus, and a tertiary lymphatic organ (31). Three doses of injection were carried out at intervals of 2 weeks without any adjuvants. Other parameters were kept defaults. The results of cytokines from C-ImmSim were analyzed for the ratio of IFN-γ to IL-10 on the 16, 30, and 40 days. The results of TGF-β, IL-2, and IL12 from C-ImmSim were analyzed on the 8, 16, and 30 days. According to the measures above, the sequences of selected differentially expressed proteins were further submitted to NCBI-CDD (https://www.ncbi.nlm.nih.gov/cdd/) and Pfam (http://pfam.xfam.org/) for conserved domains prediction.

### Evaluation of Physicochemical Properties

ProtParam (https://web.expasy.org/protparam/) was operated to determine the physicochemical properties. Protein sequences were submitted to this server to compute parameters, such as molecular weight, instability index, aliphatic index, half-life, theoretical pI, and negatively and positively charged residues.

### Tertiary Structure Prediction, Refinement, and Assessment

After the predictions of conserved domains and physicochemical properties, some differentially expressed proteins were considered as vaccine candidates. I-TASSER server (http://zhanglab.ccmb.med.umich.edu/I-TASSER) developed by Yang Zang lab was utilized to develop the tertiary structure of vaccine candidates, based on multiple threading alignments and iterative structural assembly simulations (32). Visual Molecular Dynamics software was used for the visualization of 3D structures. The tertiary structures from I-TASSER were subjected to the GalaxyRefine server (http://galaxy.seoklab.org/cgi-bin/submit.cgi?type=REFINE) for refinement. GalaxyRefine server outputs five models, of which model 1 is generated by structural perturbation and models 2-5 are produced by deeper secondary structural elements and perturbations of the loop (33). To confirm the effect of refinement, the tertiary structures from I-TASSER and GalaxyRefine were validated by the SWISS-MODEL server (https://swissmodel.expasy.org/assess) and ProSA-web (https://prosa.services.came.sbg.ac.at/prosa.php) (34). SWISS-MODEL server can calculate a Ramachandran plot that indicates favored backbone dihedral angles for each amino acid residue in protein structure. ProSA-web can produce Z-score that indicates overall model quality.

### Molecular Docking of Vaccine Candidates and Toll-Like Receptor 4

To investigate the interaction between vaccine candidates and Toll-like receptor 4 (TLR 4, PDB ID: 3FXI) with myeloid differentiation factor 2 (MD2), molecular docking was carried out using Cluspro 2.0 server (https://cluspro.bu.edu/login.php?redir/queue.php). This server is a major available tool for docking and it performs stiff docking by producing thousands of various conformations, calculating the lowest energy of clustering *via* the root mean square deviation (RMSD), and refining subjected structures (35). Here, TLR4/MD2 acted as a receptor, and vaccine candidates worked as ligands.

### Molecular Dynamics Simulation

The stability of the complexes of TLR4/MD2 and vaccine candidates was investigated with the help of MD simulation. MD simulation was performed by Gromacs 2019.6 software in CHARMM36 force field and solvated system (TIP3P water and Na+ as neutralizing counter ions). LINCS algorithm was used to constrain Covalent bonds involving hydrogen atoms and electrostatic interactions were treated with particle-mesh Ewald employing a real-space cutoff of 10 Å. To avoid steric clashes, the steepest descent algorithm approach was used for energy minimization. During the equilibration phase (100ps), MD simulation was performed for 50ns in 300K temperature and 1 bar pressure, and the stability of complexes was analyzed in terms of root mean square deviation (RMSD) and root mean square fluctuation (RMSF) (19, 30).

### Codon Optimization and *In Silico* Cloning

To make sure the expression of vaccine candidates in *Ecoil* cells, the reverse translation and codon optimization were carried out using the JCAT server (http://wwwjcat.de/) (2, 19). This server provides the results including codon optimization, codon adaptive index (CAI), and GC content. The cDNA sequences of vaccine candidates with *XhoI* and *BamH*I restriction sites were inserted into the pET32a vector for further experiments.

### Statistical Analysis

Statistical analyses were performed by IBM SPSS Statistics 22 version. The differences were evaluated using Student’s *t*-test, and the significant difference was designed as asterisks (*P<0.05, **P<0.01, ***P<0.001).

## Results

### Parasitic Infection *In Vitro*

**Figure 1:**
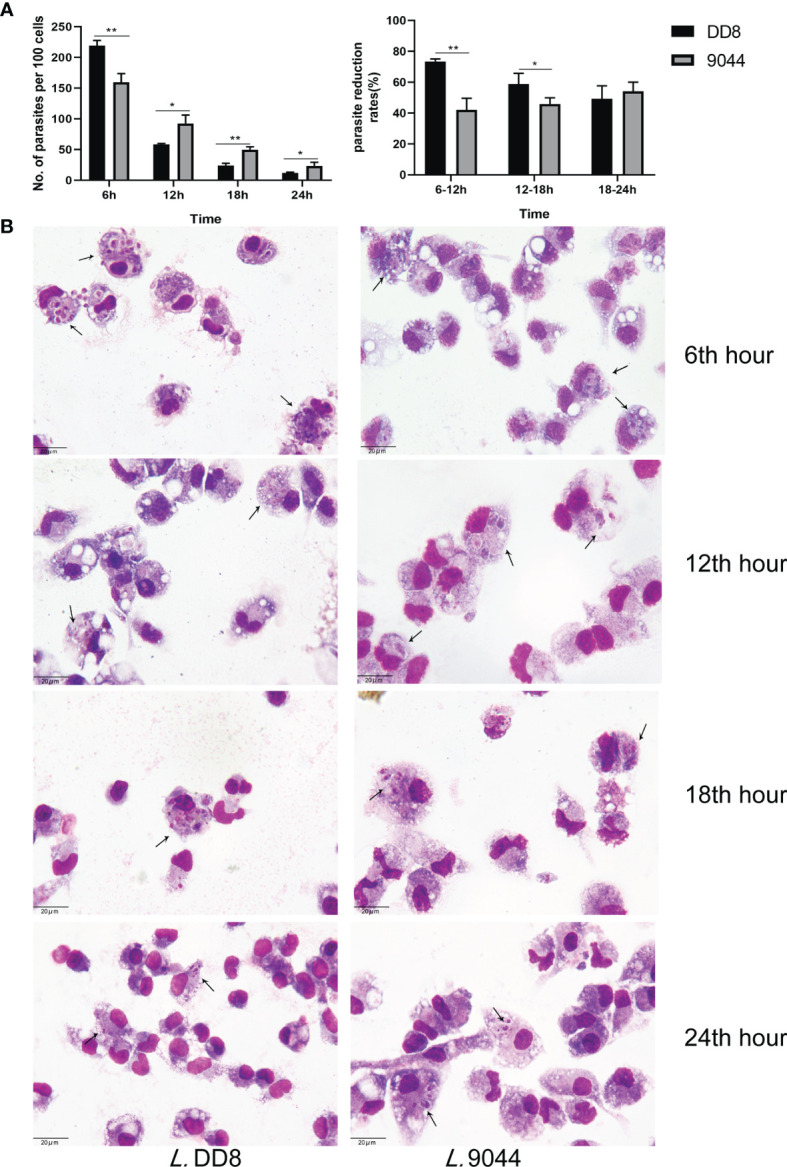
Parasite burdens of *L*. DD8 and *L*. 9044. Peritoneal macrophages were respectively infected with promastigotes (*L*. DD8 and *L*. 9044). The cells were stained by Wright’s staining for the evaluation of parasite burdens at 6, 12, 18, and 24 h. **(A)** Parasites were counted per 100 cells under 1000 power magnification. The number of parasites inside cells at each time point were presented and the ratio of burden reduction was calculated. **(B)** The cells were stained by Wright’s staining at 6, 12, 18, and 24 h, and parasites were marked using arrows. Statistical analyses were performed by IBM SPSS Statistics 22 with Student’s *t*-test, and the significant difference was designed as asterisks (*P<0.05, **P<0.01).

To confirm the difference between *L*. DD8 and *L*. 9044 in resistance to immune clearance, peritoneal macrophages were respectively co-cultured with promastigotes (*L*. DD8 and *L*. 9044) and were stained by Wright’s staining for the evaluation of parasitic load (**Figure 1** and **Supplementary Table 1**). Parasite burdens per 100 cells were estimated at 6, 12, 18, and 24 h post-infection. The loads of parasite (per 100 cells) of *L*. DD8 were more than those of *L*. 9044 at 6 h, but the burdens of *L*. DD8 strain were less than those of *L*. 9044 at 12 h, 18, and 24 h. From 6 to 12 h and 12 to 18h, the parasite reductions from *L*. DD8 were significantly more than those of *L*. 9044 (**Figure 1A**). From the results of cellular stained by Wright’s staining (**Figure 1B**), the burdens of *L*. DD8 were kept less than those of *L*. 9044 at 12, 18, and 24 h.

### Differentially Expressed Proteins Analysis

**Figure 2:**
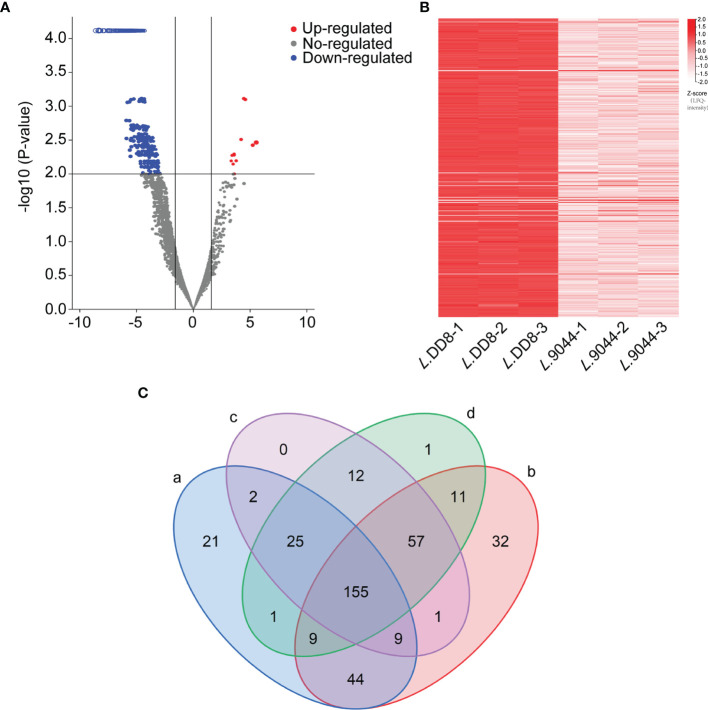
The Differentially expressed proteins analysis between *L*. DD8 and *L*. 9044 and the prediction of antigenicity, allergenicity, and human and mouse homologous proteins. **(A)** Volcanic plot map of differentially expressed proteins of *L*. 9044 vs *L*. DD8. **(B)** Heatmap of differentially expressed proteins of *L*. 9044 vs *L*. DD8. **(C)** a: The number of differentially expressed proteins having antigenic indexes > 0.5 was 266. b: The number of differentially expressed proteins that were not allergenic was 318. c: The number of differentially expressed proteins dissimilar to human proteins was 261. d: The number of differentially expressed proteins dissimilar to mouse proteins was 271.

There were 396 differentially expressed proteins between *L*. DD8 and *L*. 9044 (**Figures 2A, B**) (**Supplementary Table 2**). Furthermore, *L*. 9044 strains had 381 down-regulated proteins and 15 up-regulated proteins compared with *L*. DD8. This suggests that there are great differences in protein expression patterns between *L*. DD8 and *L*. 9044. Some of these differentially expressed proteins may enable *L.* 9044 with more virulence and act as antigenic proteins in *Leishmania donovani*.

### Prediction of Antigenicity, Allergenicity, and Human and Mice Homologous Proteins

Through the analysis of differentially expressed proteins, 396 proteins were chosen. As shown in **Figure 2C**, the antigenicity, allergenicity, and human and mouse dissimilarity of 396 differentially expressed proteins were respectively predicted. The 266 proteins were owning antigenic indexes > 0.5, and 318 proteins were not allergenic. After the blastp of sequence, the proteins dissimilar to human and mouse were 261 and 271, respectively. Differentially expressed Proteins that were simultaneously included in the analysis results of antigen index, allergen, and blastp were 155 and selected for epitope prediction (**Supplementary Table 3**).

### MHC II, IFN-γ, and MHC I Epitope Prediction

**Figure 3:**
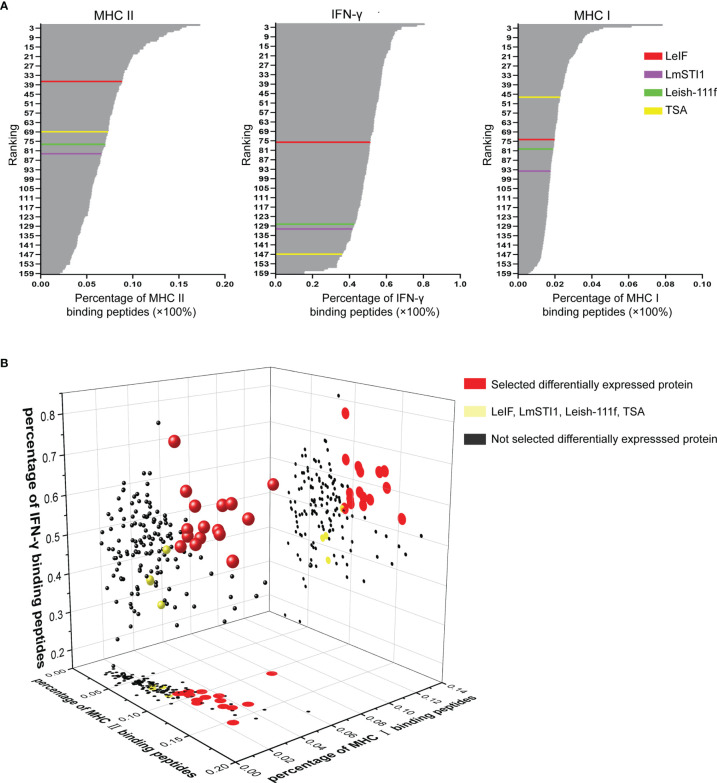
MHC II, IFN-γ, and MHC I epitope prediction. **(A)** According to the percentage of MHC II, IFN-γ, and MHC I binding peptides, 155 differentially expressed proteins and control groups (LeIF in red color, LmSTI1 in purple, Leish-111f in green color, and TSA in yellow color) were ranked respectively. **(B)** The percentages of MHC II, IFN-γ, and MHC I binding peptides were analyzed together to rank the 155 differentially expressed proteins and the results were represented as 3D scatter diagram. The 155 spheres were representative of 155 differentially expressed proteins. The 16 red spheres stranded for 16 differentially expressed proteins of which percentages of MHC II, IFN-γ, and MHC I epitopes prediction were all greater than those in the control groups (yellow sphere). The differentially expressed proteins which failed to be equipped with all the percentages greater than those of the control groups were marked as black. The plots on planes (MHC II-MHC I and MHC II-IFN-γ binding peptide percentage axis) were the projection of spheres.

The 155 differentially expressed proteins along with control groups (LeIF, LmSTI1, TSA, and Leish-111f) were employed to predict MHC II, IFN-γ, and MHC I epitope respectively. Meanwhile, the percentages of binding peptides were ranked (**Figure 3A**). In MHC II epitope prediction, the average percentage of 27 alleles binding peptides of LeIF was 8.8% and more than those of the other three control groups (TSA 7.3%, Leish-111f 7.0%, and LmSTI1 6.6%). The percentages (from 8.9% to 17.3%) of 36 differentially expressed proteins were more than that of LeIF. These proteins ranked superior to LeIF. In IFN-γ epitope prediction, LeIF (51.4%) had the highest percentage in control groups (TSA 36%, Leish-111f 42.7% and LmSTI1 41.7%). Seventy-five differentially expressed proteins were having percentages from 51.6% to 80.5% and ranked superior to LeIF (51.4%). As for MHC I epitope prediction, the average percentage of TSA was 2.3%, ranking highest in control groups at 47th (LeIF 2.0%, Leish-111f 1.9%, and LmSTI1 1.8%). The average percentages of 46 differentially expressed proteins (from 2.3% to 7.9%) were greater than TSA and ranked higher than it. Based on the above results, there were 16 differentially expressed proteins of which percentages of MHC II, IFN-γ, and MHC I epitopes prediction were all greater than those in the control groups (**Figure 3B**). These 16 proteins were suitable for vaccine candidates and taken for the next analysis. The raw dates of epitope prediction please refer to **Supplementary Table 4-1**, **4-2**, **4-3** (Please note the **Supplementary Material Section**) for MHC II prediction, **Supplementary Table 5-1, 5-2, 5-3** for IFN-γ prediction, **Supplementary Table 6** for MHC I prediction.

### The Evaluation of the Ratio of IFN-γ to IL-10 and Conserved Domain Prediction

**Figure 4:**
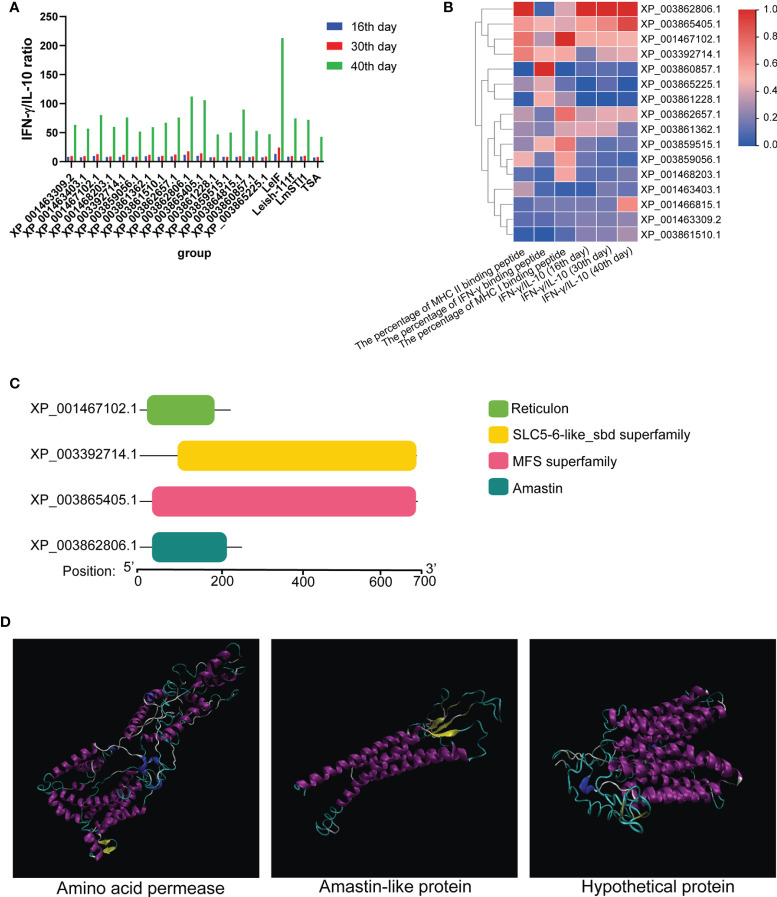
The value of IFN-γ/IL-10 and conserved domain. **(A)** The values of IFN-γ and IL-10 from 16 differentially expressed proteins were predicted using C-IMMSIM to evaluate the ratio of IFN-γ to IL-10 on 16, 30, and 40 days. **(B)** Combining with the value of IFN-γ/IL-10 and the percentage of MHC II, IFN-γ, and MHC I epitopes, 16 differentially expressed proteins were analyzed and represented as a heat map. **(C)** Amino acid permease (XP_003392714.1), amastin-like protein (XP_003862806.1), the hypothetical protein (XP_003865405.1) and papLe22 protein (XP_001467102.1) were predicted their conserved domain. Amino acid permease (residue 99-696), amastin-like protein (residue 37-206), the hypothetical protein (residue 37-690), and papLe22 protein (residue 20-189) shared a similar conserved domain with SLC5-6-like sbd family, amastin family, major facilitator superfamily (MFS) and reticulon family, respectively. **(D)** Refined 3D structures of vaccine candidates (alpha helix: purple; extended stands: yellow; beta turn: cyan; random coil: white).

To determine the type of immune response, 16 differentially expressed proteins were applied to C-IMMSIM for the evaluation of ratio of IFN-γ to IL-10 on 16, 30, and 40 days (**Figure 4A**). TGF-β, IL-12, and IL-2 were also analyzed by C-IMMSIM (**Supplementary Figure 1**) and the raw results of cytokine predicted by C-IMMSIM please refer to **Supplementary Figure 2**. IFN-γ/IL-10 value from every protein increased over time and was the highest at 40 days. LeIF, XP_003862806.1, and XP_003865405.1 had a greater value than other differentially expressed proteins and control groups on 16, 30 and 40 days. The values of IFN-γ/IL-10 and the percentages of MHC II, IFN-γ, and MHC I epitope were combined for analysis to determine the differentially expressed proteins not only full of MHC II, IFN-γ, and MHC I epitopes but also possessing a better ability to cause the higher ratios of IFN-γ/IL-10. According to **Figure 4B**, 16 differentially expressed proteins along with the values of IFN-γ/IL-10 and the percentages of MHC II, IFN-γ, and MHC I binding peptide were analyzed using heat map clustering. Differentially expressed proteins, amastin-like surface protein-like protein (amastin-like protein) (XP_003862806.1), a hypothetical protein (XP_003865405.1), 22 kDa potentially aggravating protein (papLe22) (XP_001467102.1) and amino acid permease (XP_003392714.1), performed well in the percentages of MHC II, IFN-γ, and MHC I binding peptide and the values of IFN-γ/IL-10 (16th, 30th and 40th). These four proteins formed one cluster at the top of the heat map. XP_003862806.1 and XP_003865405.1 had the lowest TGF-β levels on 8 and 16 days. XP_001467102.1 and XP_003392714.1 had low levels of TGF-β on 8, 16, and 30 days (**Supplementary Figure 1A**). On 16 days, these four proteins had high levels of IL-2 and IL-12, and had more IL-2 than the other eleven proteins (including TSA, LmSTI1, and Leish-111f) and more IL-12 than the other twelve proteins (including TSA, LmSTI1, and Leish-111f) (**Supplementary Figures 1B, C**). These four proteins were more desirable proteins as vaccine, which contributed to predicting the conserved domains of these four proteins. Amino acid permease, amastin-like protein, the hypothetical protein (XP_003865405.1), and papLe22 protein shared similar motifs with the SLC5-6-like sbd family, amastin family, major facilitator superfamily (MFS), and reticulon family, respectively (**Figure 4C**). SLC5-6-like sbd family and MFS participate in transportation for metabolism (36–38). The amastin family is responsible for interaction with hosts and invasion (39). The reticulon family is involved in protein synthesis. Amino acid permease, amastin-like protein, and the hypothetical protein (XP_003865405.1) were employed as vaccine candidates.

### Evaluation of Physicochemical Properties

With the help of ProtParam, three vaccine candidates [amino acid permease, amastin-like protein, and the hypothetical protein (XP_003865405.1)] were computed for their physicochemical properties. Amino acid permease contained 605 amino acids, 65.5KDa molecular weight, 7.15 theoretical pI, and 42 negatively and positively charged residues. Its estimated half-lives were 30 hours (mammalian reticulocytes, *in vitro*), >20 hours (yeast, *in vivo*), and >10 hours (*Escherichia coli*, *in vivo*). The instability index (II) was calculated as 35.97, indicating that it is a stable protein (value < 40 is classified as stability). The aliphatic index was 95.92 and showed more thermostability (the higher aliphatic index, the more stable in a broad range of temperature.) (19). As for amastin-like protein, it contained 222 amino acids, 24.5 KDa molecular weight, 6.41 theoretical pI, 18 negatively and 17 positively charged residues. Its estimated half-lives were 30 hours (mammalian reticulocytes, *in vitro*), >20 hours (yeast, *in vivo*), and >10 hours (*Escherichia coli*, *in vivo*). The instability index (II) was 32.61, indicating that it is a stable protein. The aliphatic index was 94.46 and showed more thermostability. The hypothetical protein (XP_003865405.1) had 607 amino acids, 66.2 KDa molecular weight, 8.52 theoretical pI, 42 negatively, and 48 positively charged residues. Its estimated half-lives were 30 hours (mammalian reticulocytes, *in vitro*), >20 hours (yeast, *in vivo*), and >10 hours (*Escherichia coli*, *in vivo*). The instability index (II) was 35.07 and suggested it is a stable protein. The aliphatic index was 111.55, indicating more thermostability.

### Tertiary Structure Prediction, Refinement, and Assessment

**Figure 5:**
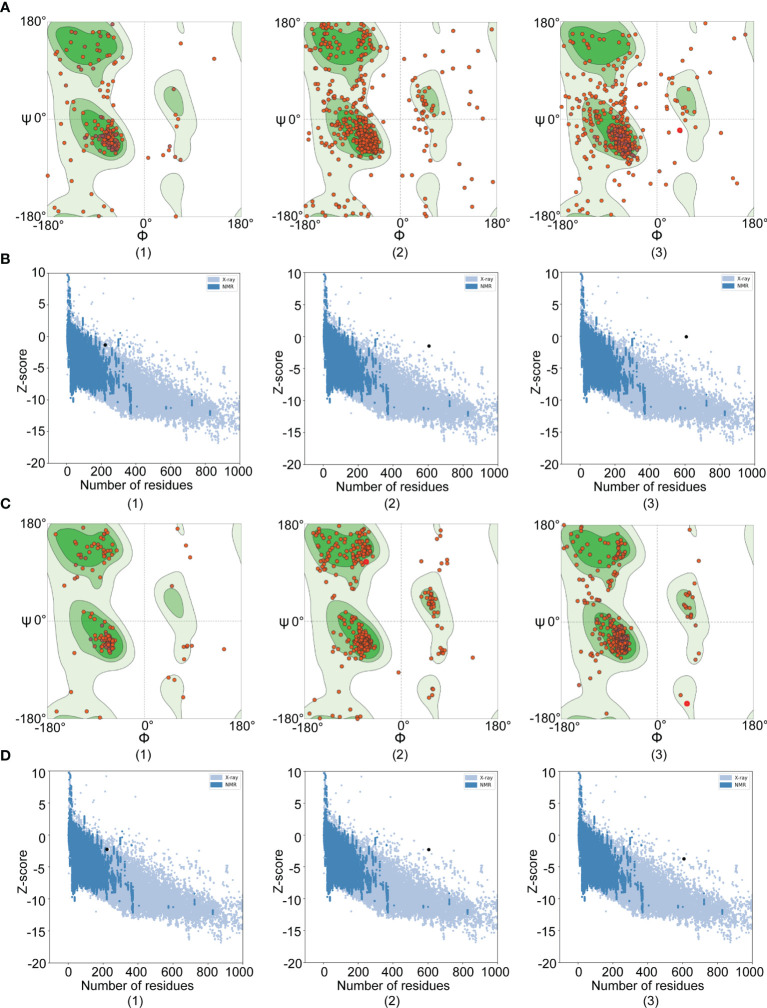
Ramachandran and Z-score plots of initial and refined 3D models. The results of Amastin-like protein, Amino acid permease, and hypothetical protein (XP_003865405.1) were respectively remarked with (1), (2), and (3). **(A)** Ramachandran plots of initial models: Amino acid permease showed 70.71% favored region and 11.83% outlier region; Amastin-like protein had 83.62% favored region and 6.90% outlier region; The hypothetical protein (XP_003865405.1) demonstrated 71.9% favored region and 14.05% outlier region. **(B)** Z-score plots of initial models: Amino acid permease, amastin-like protein, and the hypothetical protein (XP_003865405.1) were -1.48, -1.32, and -0.1 value, respectively. **(C)** Ramachandran plots of refined models: Amino acid permease showed 91.21% favored region and 1.49% outlier region; Amastin-like protein had 90.45% favored region and 2.27% outlier region; The hypothetical protein (XP_003865405.1) demonstrated 92.73% favored region and 1.65% outlier region. **(D)** Z-score plots of refined models: Amino acid permease, amastin-like protein, and the hypothetical protein (XP_003865405.1) were -2.27, -2.26, and -3.6 value, respectively.

Tertiary structure models of amino acid permease, amastin-like protein, and the hypothetical protein (XP_003865405.1) were generated with the help of I-TASSER. To enhance the quality of the 3D structure, the GalaxyRefine server was employed to refine the selected initial models and generated five refined models. The selected refined models were amino acid permease (GDT-HA 0.99, RMSD 0.272, MoProbity 2.159, Clash score 14.1, Poor rotamers 0.6 and Ramafavored 91.7%), amastin-like protein (GDT-HA 0.98, RMSD 0.254, MoProbity 2.242, Clash score 17.8, Poor rotamers 0.5 and Ramafavored 91.8%) and the hypothetical protein (XP_003865405.1) (GDT-HA 0.97, RMSD 0.319, MoProbity 2.453, Clash score 32.5, Poor rotamers 0.6 and Ramafavored 92.7%). Visualizations of the refined 3D structure were shown in **Figure 4D**. Initial and refined models were subjected to SWISS-MODEL for Ramachandran plot analysis. More residues of the model in Ramachandran favored region and less in outlier region indicate desirable models. Before refinement, Ramachandran plot results from initial models revealed amino acid permease (70.71% favored region and 11.83% outlier region), amastin-like protein (83.62% favored region and 6.90% outlier region), and the hypothetical protein (XP_003865405.1) (71.9% favored region and 14.05% outlier region) (**Figure 5A**). Refined models were found that the favored and outlier regions were 91.21% and 1.49% (amino acid permease), 90.45% and 2.27% (amastin-like protein) and 92.73% and 1.65% [hypothetical protein (XP_003865405.1)] (**Figure 5C**). ProSA-web provides Z-score that indicates overall model quality. Z-score close to the regions that X-ray and NMR produced indicates better structure (40). As for amino acid permease, the Z-scores of initial and refined model were respectively -1.48 and -2.27. The Z-scores from amastin-like protein were -1.32 (initial model) and -2.26 (refined model). The Z-scores of the hypothetical protein (XP_003865405.1) were -0.1 (initial model) and -3.6 (refined model) (**Figures 5B, D**). According to the results of the Z-score, the lower values were closer to the regions of X-ray and NMR.

### Molecular Docking of Vaccine Candidates and Toll-Like Receptor 4

**Figure 6:**
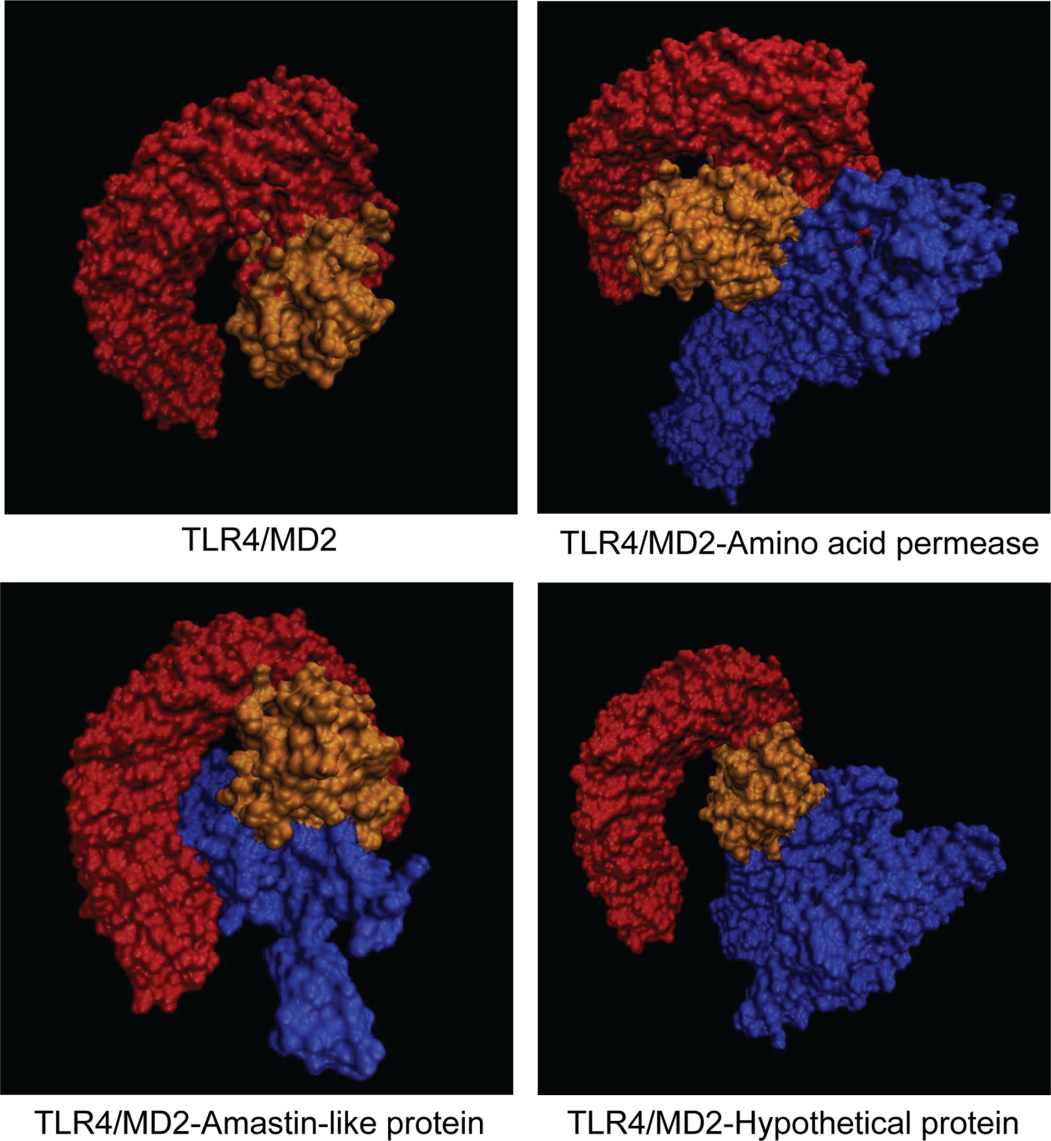
Representation of docked complexes between TLR4/MD2 and vaccine candidates. TLR4 and MD2 were defined as orange and yellow, respectively. Three vaccine candidates including amino acid protein, amastin-like protein, and the hypothetical protein (XP_003865405.1) were marked in blue.

Molecular docking was performed by Cluspro 2.0 server to investigate the interaction between vaccine candidates and TLR4/MD2 (PDB ID: 3FXI) (**Figure 6**). TLR4/MD2 and three vaccine candidates were respectively defined as receptor and ligands. Cluspro 2.0 server provides 30 models based on the number of the cluster with low energy structure. More clusters in docked complex indicate a better encounter complex (35). To gain available docked complexes, models with 32 clusters and -1301 lowest energy (amino acid permease), 58 clusters and -1236.5 lowest energy (amastin-like protein), and 52 clusters and -1232.9 lowest energy [hypothetical protein (XP_003865405.1)] were taken for molecular dynamics simulation.

### Molecular Dynamics Simulation

**Figure 7:**
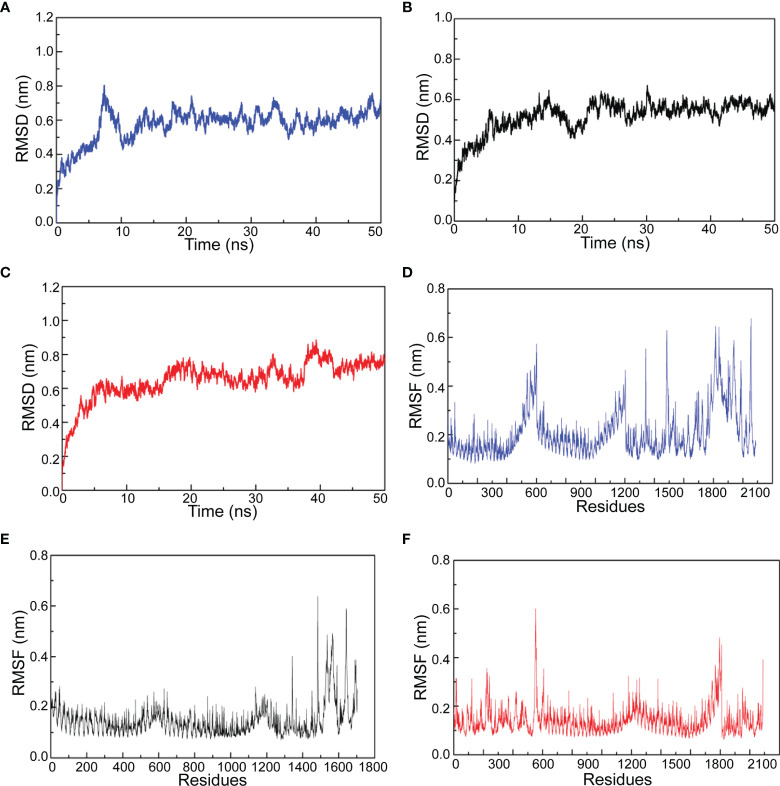
The results of molecular dynamics simulation. **(A)** Amino acid permease and TLR4/MD2 complex RMSD. **(B)** Amastin-like protein and TLD4/MD2 complex RMSD. **(C)** Hypothetical protein (XP_003865405.1) and TLR4/MD2 complex RMSD. **(D)** Amino acid permease and TLR4/MD2 complex RMSF. **(E)** Amastin-like protein and TLD4/MD2 complex RMSF. **(F)** Hypothetical protein (XP_003865405.1) and TLR4/MD2 complex RMSF.

Molecular dynamics simulation provides chances to investigate the stability of docked complex between TLR4/MD2 and vaccine candidates. After the equilibration phase, MD simulation was performed for 50 ns in 300K temperature and 1 bar pressure. Root mean square deviation (RMSD) shows the fluctuation of the overall structure of the docked complex and root medium square fluctuation (RMSF) represents the fluctuation of amino acid from the docked complex. As shown in **Figures 7A–C**, the RMSD of amastin-like, amino acid permease, and the hypothetical protein (XP_003865405.1) complexes had obvious fluctuation during 0-10 ns simulation. After 10 ns, the RMSD of amastin-like, amino acid permease, and the hypothetical protein (XP_003865405.1) complexes were kept at 0.5 nm, 0.6 nm, and 0.7nm, indicating their stable conformation. From the results of the RMSF plot, the RMSF fluctuation of amastin-like complex appeared at 1500-1800 amino acid, showing high flexibility (**Figure 7E**). The residues 0-1500 of the amastin-like complex had stable RMSF, indicating low flexibility. Amino acid permease complex had low RMSF value at 0-400 and 700-1000 amino acid, suggesting these residues had low flexibility (**Figure 7D**). Conversely, the residues 400-700 and 1000-2100 of Amino acid permease complex had greater RMSF value, showing large flexibility. As for the hypothetical protein (XP_003865405.1) complex, the residues 0-650, 1100-1400, and 1650-2100 were significantly flexible because of obvious RMSF fluctuation, and the rest had stable RMSF fluctuation, suggesting low flexibility (**Figure 7F**).

### Codon Optimization and *In Silico* Cloning

**Figure 8:**
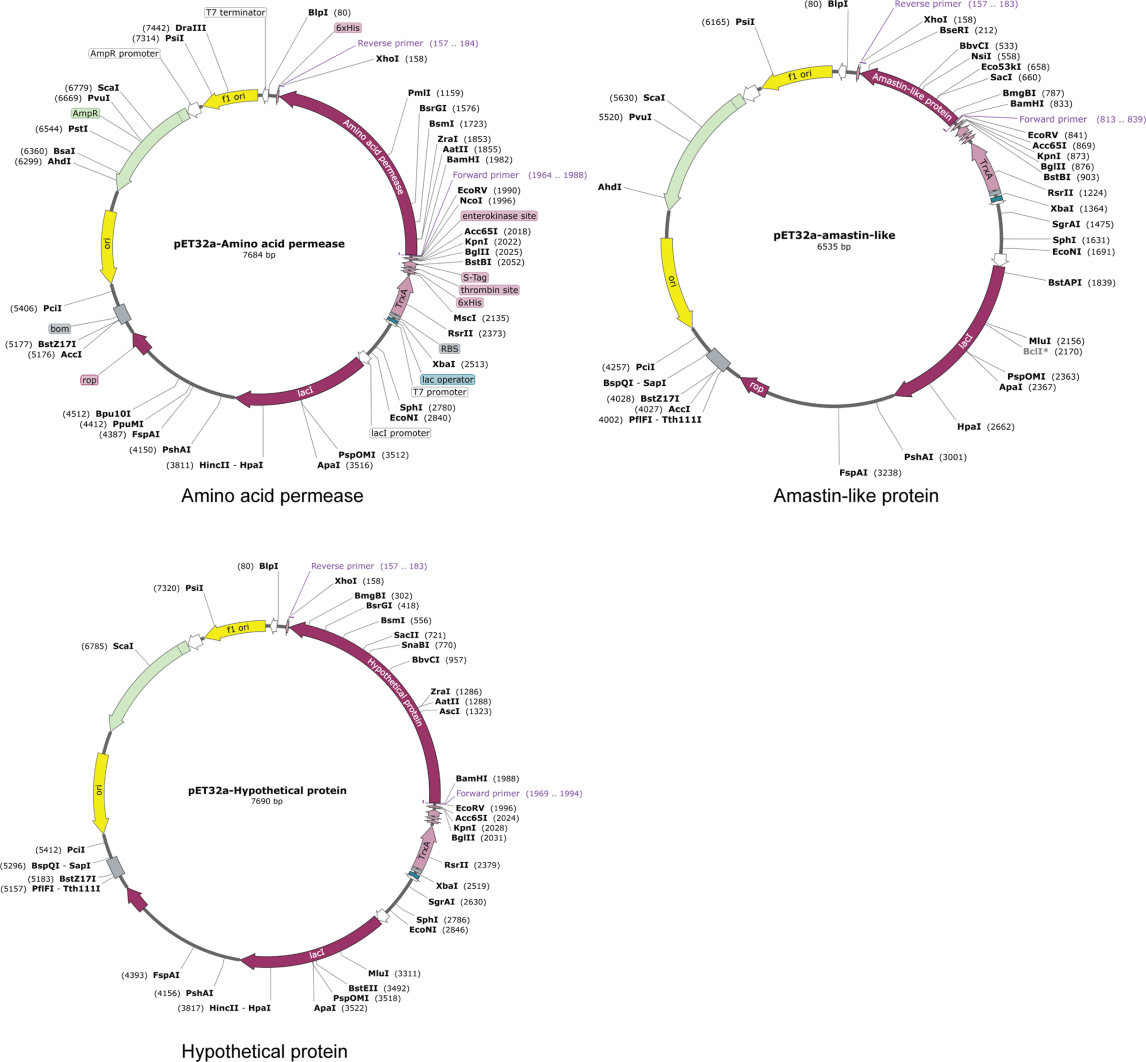
*In silico* cloning. The coding sequences of amino acid permease, amastin-like protein, and the hypothetical protein (XP_003865405.1) were cloned into the pET32a (+) expression vector and represented in the red region. The inserts were added with *XhoI* and *BamH*I restriction sites.

The reverse translations and codon optimizations of amino acid permease, amastin-like, and the hypothetical protein (XP_003865405.1) were performed using the JCAT server to investigate the stability of expression in *Ecoil K12* cells. After reverse translations and codon optimizations, codon adaptation index (CAI) and GC content were analyzed. The CAI of amino acid permease protein was 1.0 which was considered as optimal expression. Its GC content was 51.81%, lying in the desirable range (30% to 70%) that is easily expressed in a suitable host (41). Amastin-like protein had 1.0 CAI and 49.47% GC and the hypothetical protein (XP_003865405.1) had 1.0 CAI and 50.87% GC. Three vaccine candidates were cloned into pET32a with *XhoI* and *BamH*I restriction sites, using Snapgene standalone software (**Figure 8**).

## Discussion

Almost existing epitope screening methodologies for vaccine against VL are classical approaches and immunoinformatics. Classical approaches synthesize overlapping peptides (usually 15-mers) to stimulate T cell clones *in vitro* and *in vivo* for mapping epitopes (42–44). This way requires lots of time and energy and may neglect some epitopes, which could be saved by immunoinformatics (42, 45). Based on mathematical algorithms, immunoinformatics is concerned with computational prediction of epitopes from proteins and contributes to a new pattern of vaccine design (42, 46). Then, epitopes predicted by immunoinformatics are submitted to further experiments *in vitro* and *in vivo* for confirmation. *Leishmania* antigenic proteins validated by experiments usually are screened for their epitopes of T and B cells by immunoinformatics (2, 30, 47, 48). However, this approach is mainly used to construct multi-epitope vaccines against VL and relies on known antigenic proteins. It is seldom supplied to discover vaccine potential from novel proteins. The differentially expressed proteins from different species (virulent and low virulent) may be associated with *leishmania* survival and offer more chances to identify novel vaccine candidates (8, 13, 14). In our studies, differentially expressed proteins were analyzed by bioinformatics to select the proteins with rich epitopes and high IFN-γ/IL-10 ratios as novel protein vaccine candidates.

In our previous studies, BALB/c mice were infected with *L*. DD8 and *L*. 9044 for 28 days, and the parasite loads of *L*. 9044 in livers and spleens were more than those of *L*. DD8 (26). Those results of parasite load from mice were consistent with that of infection in peritoneal macrophages, suggesting that *L. 9044* had more virulence than *L. DD8.* The 396 differentially expressed proteins between *L*. 9044 (virulent) and *L*. DD8 (low virulent) were explored *in silico* to screen the new vaccine candidates in our present studies. The 396 differentially expressed proteins from LC-MS/MS analysis were submitted to analyze their antigenicity, allergenicity, and human and mouse homologous proteins and then 155 differentially expressed proteins were selected for MHC II, IFN-γ and MHC I binding peptide prediction. Subsequently, the 16 differentially expressed proteins suitable for vaccine were selected for the analyses of IFN-γ/IL-10 ratio. Four differentially expressed proteins, having large ratio of IFN-γ/IL-10 ratio and high percentage of MHC II, IFN-γ, and MHC I, were considered as desirable proteins as vaccine and were taken for conserved domain analysis. Finally, three differentially expressed proteins, amino acid permease, amastin-like protein, and the hypothetical protein (XP_003865405.1) were selected as vaccine candidates for future studies.

Early studies predicted the T cell epitopes of differentially expressed protein from promastigotes and amastigotes for vaccine (49). Shubhranshu et al. focused on the promastigote differentially expressed proteins between avirulent and virulent strains and screened the protein which may work as a virulence factor to study its immunogenicity and immunoprotection (50). Mohammad et al. found that some upregulated proteins from virulent *Leishmania donovani* were more capable of restraining MAPK and PI3K signaling, but some downregulated proteins also have immune protection and virulence-related properties, such as 60S acidic ribosomal protein P2 and tryparedoxin peroxidases (51). In our studies, the differentially expressed proteins of *L*. 9044 and *L*. DD8, including both upregulated and downregulated proteins, were used to screen ideal vaccine candidates.

It is acknowledged that parasites can replicate in hosts due to inactive macrophages. CD4+ Th1 cells are of great significance to IFN-γ and TNF-α secretion and are considered to have a vital role in parasitic clearance. CD8+ T cells also secret IFN-γ and TNF-α to participate in the resistance to *leishmania* reinfection, although some studies showed that they were not essential for the control of primary infection (52). That *Leishmania* establishes intracellular residence makes it possible that humoral immune response is not as effective as a cellular immune response (43). In conclusion, *Leishmania* desirable vaccine candidates should trigger a strong Th1 immune response where CD4+ and CD8+ T cells recognize the epitopes of MHC I and MHC II and macrophages activated by IFN-γ eliminate parasites. Therefore, our studies predicted the MHC I, MHC II, and IFN-γ epitopes of differentially expressed protein between *L*. 9044 and *L*. DD8.

Four vaccine candidates, LeIF (the *Leishmania* elongation initiation factor), TSA (Thiol-specific antioxidant), LmSTI-1 (*Leishmania major* homologue to eukaryotic stress-inducible protein), and Leish-111f, have been tested in clinical trials and showed the desirable immunogenicity and safety in healthy volunteers, seronegative, and seropositive *Leishmania* patients (7). LeIF can stimulate vaccinated splenocytes to produce IFN-γ and promote Th1 immune response, and it gives protection against *L*. major because of its T cell epitopes (22). TSA can be processed through the antigen processing of MHC I and MHC II pathways. Its DNA vaccine favors the production of IFN-γ-secreting-CD4+ cells and CD8+ cells, which supports the resistance to *Leishmania* (23). LmSTI-1 also induces Th1 immune response along with high levels of IFN-γ and IgG2a and provides excellent protection against *L. major* (24). Leish-111f induces increased CD4+ cells that produce IFN-γ, IL-2, and TNF-α, which confers desirable protection against visceral leishmaniasis with significant reductions in parasite loads (25). Those performances of LeIF, TSA, LmSTI-1, and Leish-111f indicate that there are effective epitopes in these vaccine candidates to develop the Th1 immune response, such as MHC I, MHC II, and IFN-γ epitopes. In summary, LeIF, TSA, LmSTI-1, and Leish-111f, the candidates in clinical trials, were employed as control groups when we analyzed the MHC I, MHC II, and IFN-γ epitopes of differentially expressed proteins.

To further select our vaccine candidates equipped with characteristics similar to or better than those proteins (control group: LeIF, TSA, LmSTI-1, and Leish-111f) that have been shown to induce a protective immune response, sixteen differentially expressed proteins of which rates of MHC I, MHC II, and IFN-γ epitopes were greater than those of control groups were chosen in our studies. IFN-γ and IL-10 are respectively Th1 and Th2 immune response signature cytokines. IFN-γ serves as monocyte-activating factor and boosts the production of pro-inflammatory cytokines, the expression of MHC II, and antigen presentation (53, 54). IFN-γ inhibits IL-10 production and the expansion of CD4+ Th2 cells, and it matters enormously to support macrophage activation in leishmanicidal state (55, 56). It has been reported that the children with high levels of IFN-γ resist *L. chagasi* infection, whereas children with low levels of IFN-γ are susceptible to VL (57, 58). IL-10 can suppress production of IFN-γ, IL-12, TNF-α, and IL-6 and downregulate Th1 responses, macrophage activation and antigen presentation by DC cells (54, 58, 59). Abundant secretion of IL-10 accompanies with symptomatic VL but absent in asymptomatic individuals (54). It has been documented that IL-10 neutralization supports *leishmania* parasite clearance (60). IFN-γ and IL-10 are used to demonstrate the levels of Th1 and Th2 immune response by the ratio of IFN-γ/IL-10 (61). Combining IFN-γ/IL-10 ratios with the rates of MHC I, MHC II, and IFN-γ epitopes, amino acid permease, amastin-like surface protein-like protein (amastin-like protein), the hypothetical protein (XP_003865405.1), and 22 kDa potentially aggravating protein (papLe22) were selected for further studying. TGF-β can induce Treg cells and suppress Th1 and Th2 development and some macrophage functions (62). TGF-β and IL-10 are inhibitory effects on the development of VL and favor the progress of *leishmania* (7, 63). IL-12 is secreted from inflammatory myeloid cells and promotes differentiation of naive CD4 T cells into IFN-γ–producing Th1 cells (64). It is considered as a vital role that contributes to parasite clearance (61). IL-2 is an essential cytokine and is required for the survival, proliferation and differentiation of CD4, CD8 and NK cells (65). It was reported that mice treated with IL-2 blocking monoclonal antibodies could not resist the infection of *L. donovani*, while the infected mice receiving exogenous IL-2 had reduced parasite loads, relative with controls (66). Comparing with control groups together with other differentially expressed proteins, these four proteins had high levels of IL-12 and IL-2 and low levels of TGF-β, which might be conducive to parasite elimination.

Amino acid permease, amastin-like protein, the hypothetical protein (XP_003865405.1), and papLe22 protein shared similar motifs with the SLC5-6-like sbd family, amastin family, major facilitator superfamily, and reticulon family, respectively. SLC5-6-like sbd family, the Solute carrier family 5 and 6-like and solute binding domain, serves as co-transporter transporting Na+, sugars, and amino acids (67, 68). For example, *Leishmania* amino acid permease 3 (AAP3) from the SLC5-6-like sbd family enables parasites to complete for L-arginine from macrophages, which results in the reduction of NO synthesis in macrophages and supports parasitic growth (36, 37). The major facilitator superfamily (MFS) has a large and diverse group of secondary transporters and MFS proteins are responsible for the transportation of ions, short peptides, amino acids, and nucleotides (38, 69). An example of MFS proteins from *Leishmania* is *Leishmania* Iron Regular 1 (LIR1) which functions in iron export to prevent the accumulation of intracellular iron for the avoidance of toxicity (70). Our amino acid permease and the hypothetical protein (XP_003865405.1) may be involved in the metabolic pathways of *Leishmania*. As for amastin, it is acknowledged that amastin, the surface glycoproteins, participates in the formation of interaction between parasites and host cell membranes (39). Infection of intraperitoneal macrophage and mice with wild type, knocking down δ-amastin, and re-expressing δ-amastin promastigotes were performed by de Paiva RM et al, and the results (increased parasite loads: wild type and re-expressing δ-amastin promastigotes, and decreased parasite burdens: knocking down δ-amastin promastigotes) suggested that amastin was required as a vital virulence for parasite intracellular multiplication and survival (39). The amastin-like protein may act as the same virulence as amastin. PapLe22 protein involved in protein synthesis has been studied for its effects of vaccine (71, 72), which causes us to stop studying it.

TLR4 primarily contributes to the immune response against *Leishmania* invasion. The interaction between the designed vaccine and TLR4 was investigated by many studies (2, 30, 34, 73). Some binding of TLR4 needs myeloid differentiation factor 2 (MD-2) as coreceptors, such as lipopolysaccharide (LPS), Chlamydia pneumonia heat shock protein 60, Respiratory Syncytial and Virus (RSV) fusion protein (74–76). Based on the evaluation of Bahareh et al. the interaction between TLR4 and vaccine may be medicated by MD-2 (2). Hence, the TLR4/MD-2 and vaccine candidates were subjected to docking and molecular dynamic simulation. The results indicated the stability and flexibility of the TLR4/MD-2-vaccine candidate complex and supported the interaction between TLR4/MD-2 and vaccine candidates. That interaction suggested that vaccine candidates may also have the function of adjuvant and may trigger TLR4 signaling to favor Th1 immune response.

In conclusion, we have selected three vaccine candidates from differentially expressed proteins between virulent and low virulent strains, considering the rates of MHC I, MHC II, and IFN-γ epitopes, the ratio of Th1/Th2 and conserved domains. The three candidates, amino acid permease, amastin-like protein, and the hypothetical protein (XP_003865405.1) will be used to inject animals for immunization and explore the protection against *Leishmania* parasites. Our studies about new vaccine candidates *in silico* can also provide references for other pathogenic vaccine designs.

## Data Availability Statement

The datasets presented in this study can be found in online repositories. The names of the repository/repositories and accession number(s) can be found below: http://proteomecentral.proteomexchange.org/cgi/GetDataset?ID=PXD017089, PXD017089.

## Ethics Statement

The animal study was reviewed and approved by Sichuan University Medical Ethics Committee (Approval Number: K2018056).

## Author Contributions

JZ, JL, QZ, JH, XL, XC, and YX performed peritoneal macrophage culture, parasitic infection and the preparation of LC-MS/MS. JZ, JL, and KH performed the blastp and the predictions of antigenic index, epitope and IFN-γ/IL-10 value. 3D structure building and refinement, docking and molecular dynamics simulation were carried out by JZ, YC, and HQ. Visualizations were conducted by JZ, KH, and SY. JZ and KH wrote the manuscript; ZZ, JL, XC, and JC reviewed the manuscript. All the authors approved this final manuscript. All authors contributed to the article and approved the submitted version.

## Funding

This work was supported by the National Natural Science Foundation of China (grant number: 81672048 to JC; grant number: 31802184 to JL; grant number: 82102425 to JH) and the Fundamental Research Funds for the Central Universities (2022SCU12027 to ZZ and 2021SCU12077 to JH).

## Conflict of Interest

The authors declare that the research was conducted in the absence of any commercial or financial relationships that could be construed as a potential conflict of interest.

## Publisher’s Note

All claims expressed in this article are solely those of the authors and do not necessarily represent those of their affiliated organizations, or those of the publisher, the editors and the reviewers. Any product that may be evaluated in this article, or claim that may be made by its manufacturer, is not guaranteed or endorsed by the publisher.

## Supplementary Material

Supplementary Table 4-1, 4-2, and 4-3 are presented in Figshare.

**Supplementary Table 4-1** | https://figshare.com/s/a63a7b5312b8aa81127c.

**Supplementary Table 4-2** | https://figshare.com/s/d098441e751382545c14.

**Supplementary Table 4-3** | https://figshare.com/s/6ed19ec57acb4860e1b2.
