# Supplementary material for: Screening Novel Vaccine Candidates for Leishmania Donovani by Combining Differential Proteomics and Immunoinformatics Analysis
Source: Front Immunol. 2022 Jun 23;13:902066. doi: 10.3389/fimmu.2022.902066 (PMC9260594; doi:10.3389/fimmu.2022.902066)
Supplement: Supplementary file 1 [file DataSheet_1.docx]

**Supplementary figure 1:** **the results of** **TGF-β, IL-2, and IL-12 from C-IMMSIM**


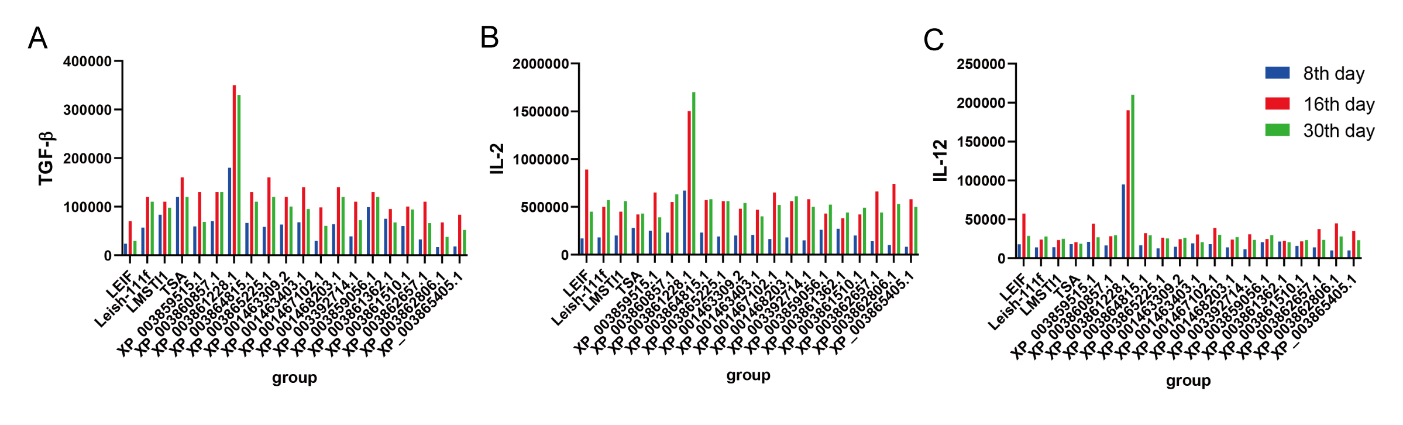


**Supplementary figure 1**. Concentration of TGF-β, IL-2, and IL-12 analyzed by C-ImmSim online server. Sixteen differentially expressed proteins were submitted to C-ImmSim and were injected for three times at intervals of 2 weeks without any adjuvant. Concentration of TGF-β (A), IL-2 (B), and IL-12 (C) on 8th, 16th, and 30th day are shown respectively.
