# Supplementary material for: Screening Novel Vaccine Candidates for Leishmania Donovani by Combining Differential Proteomics and Immunoinformatics Analysis
Source: Front Immunol. 2022 Jun 23;13:902066. doi: 10.3389/fimmu.2022.902066 (PMC9260594; doi:10.3389/fimmu.2022.902066)
Supplement: Supplementary file 2 [file DataSheet_2.docx]

**Supplementary figure 2: the cytokines of** **immune simulations from** **16 kinds of different expression protein, LmST1, LeIF, Leish-111f and TSA (raw data).**

C-ImmSim online server at (http://kraken.iac.rm.cnr.it/C-IMMSIM/) was applied for immune simulations. Sixteen kinds of different expression protein, LmST1, LeIF, Leish-111f and TSA without any adjuvant were injected for three times at intervals of 2 weeks. The results (raw data) of cytokines are shown as follow:

**XP_001463309.2**

**
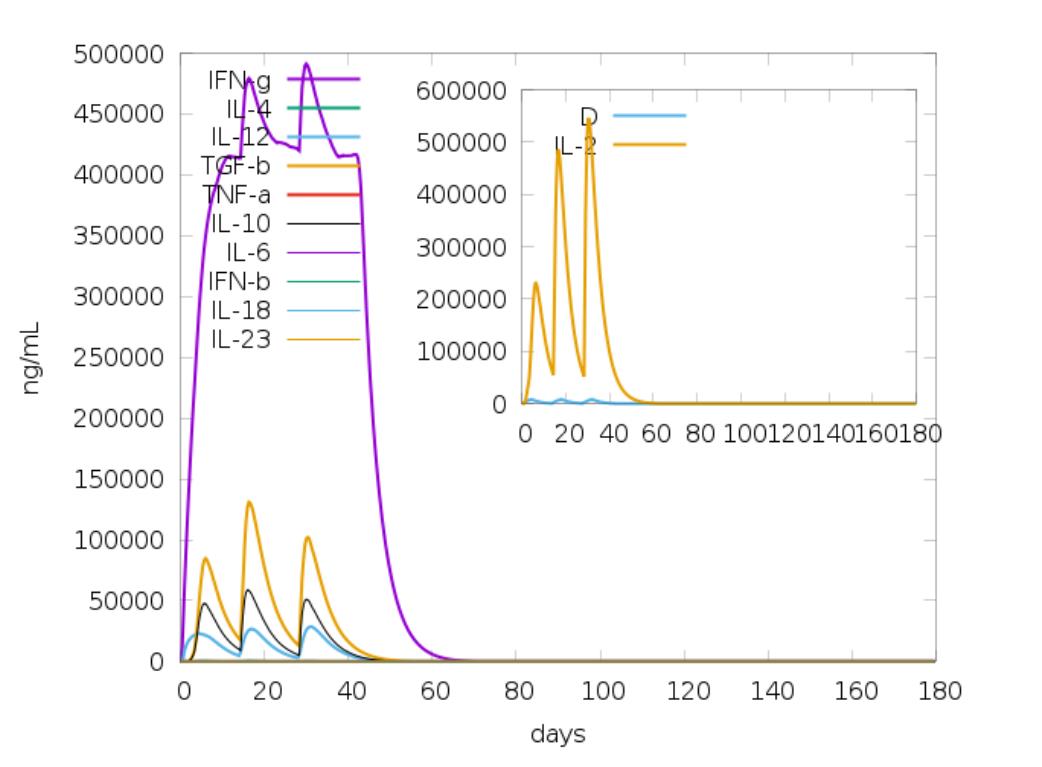
**

**Supplementary figure 2-1.** Concentration of cytokines and interleukins analyzed by C-ImmSim online server**.** XP_001463309.2 protein without any adjuvant was injected for three times at intervals of 2 weeks. IFN-gamma (IFN-γ), TGF-b (TGF-β), IL-10, and IL-12 are shown in purple line, yellow thick line, black line, and blue thick line respectively. IL-2 and danger signal (D) are presented in yellow thick line and blue thick line in the insert plot respectively.

**XP_001463403.1**


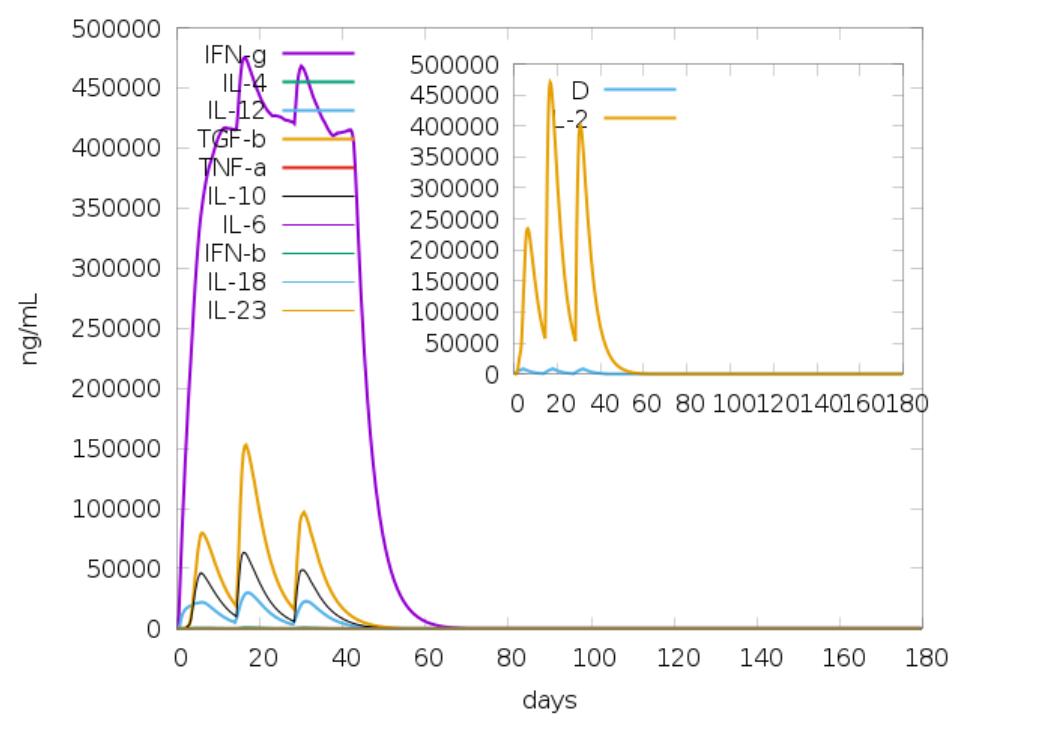


**Supplementary figure 2-2.** Concentration of cytokines and interleukins analyzed by C-ImmSim online server**.** XP_001463403.1 protein without any adjuvant was injected for three times at intervals of 2 weeks. IFN-gamma (IFN-γ), TGF-b (TGF-β), IL-10, and IL-12 are shown in purple line, yellow thick line, black line, and blue thick line respectively. IL-2 and danger signal (D) are presented in yellow thick line and blue thick line in the insert plot respectively.

**XP_001467102.1**


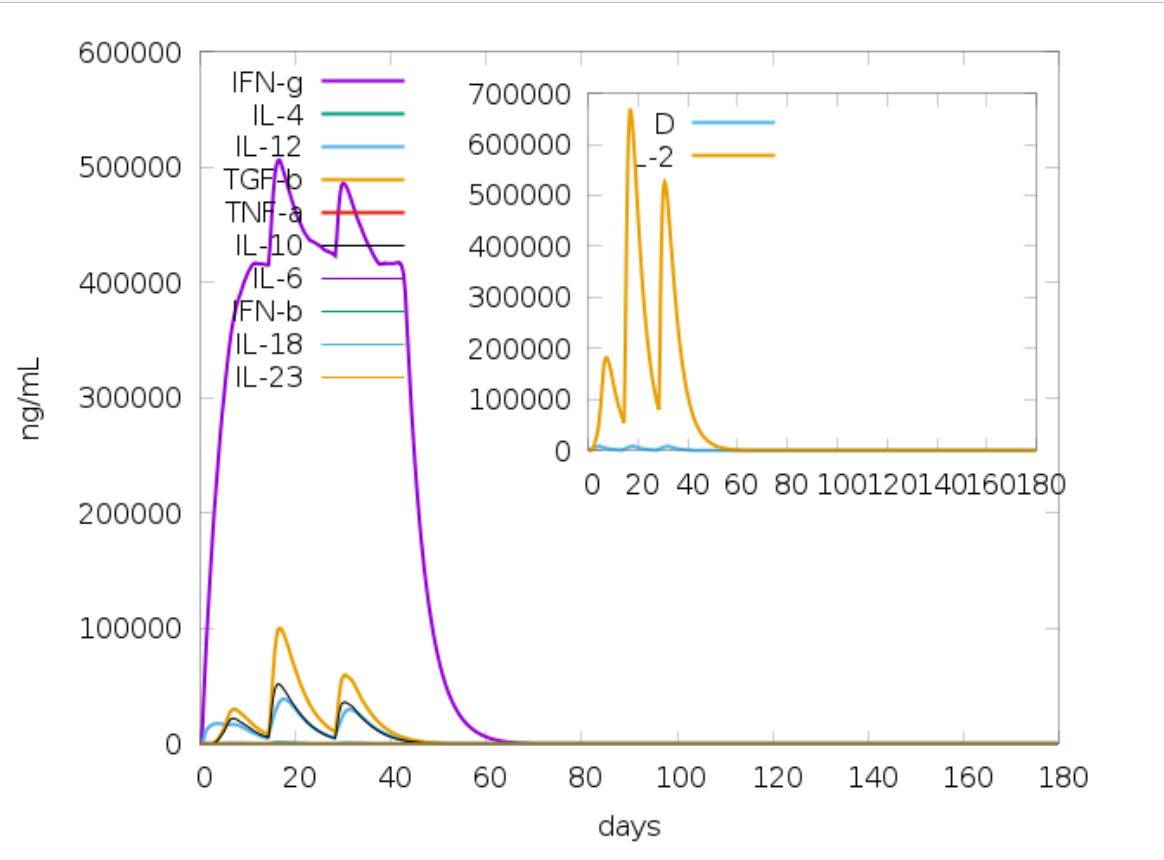


**Supplementary figure 2-3.** Concentration of cytokines and interleukins analyzed by C-ImmSim online server**.** XP_001467102.1 protein without any adjuvant was injected for three times at intervals of 2 weeks. IFN-gamma (IFN-γ), TGF-b (TGF-β), IL-10, and IL-12 are shown in purple line, yellow thick line, black line, and blue thick line respectively. IL-2 and danger signal (D) are presented in yellow thick line and blue thick line in the insert plot respectively.

**XP_001468203.1**


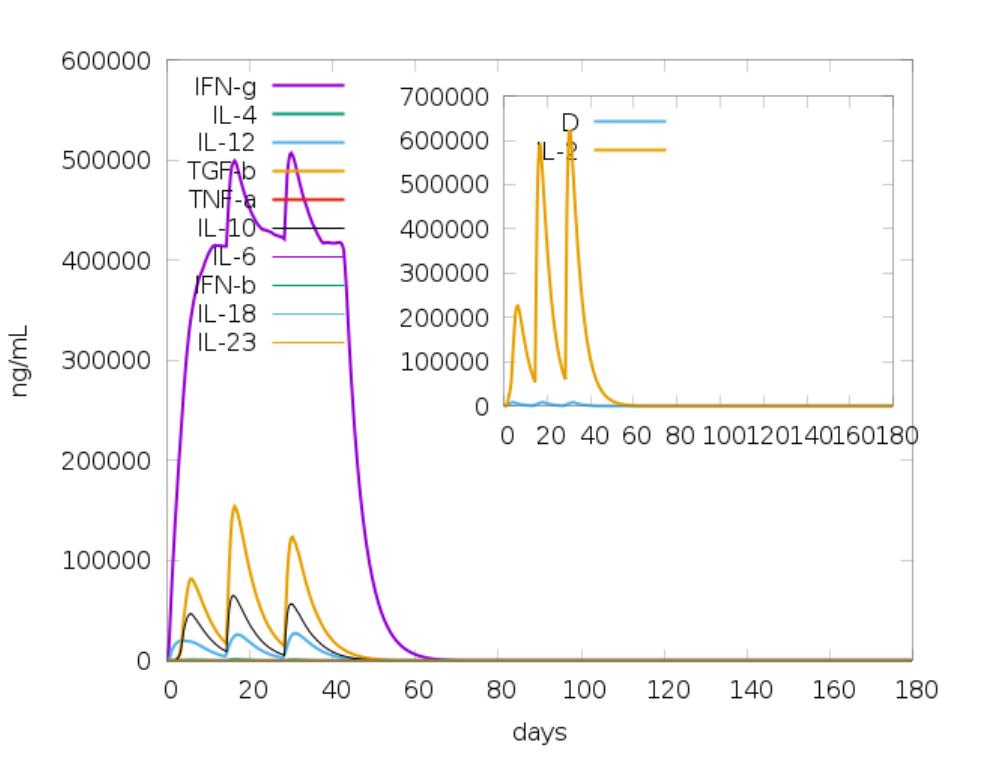


**Supplementary figure 2-4.** Concentration of cytokines and interleukins analyzed by C-ImmSim online server**.** XP_001468203.1 protein without any adjuvant was injected for three times at intervals of 2 weeks. IFN-gamma (IFN-γ), TGF-b (TGF-β), IL-10, and IL-12 are shown in purple line, yellow thick line, black line, and blue thick line respectively. IL-2 and danger signal (D) are presented in yellow thick line and blue thick line in the insert plot respectively.

**XP_003392714.1**


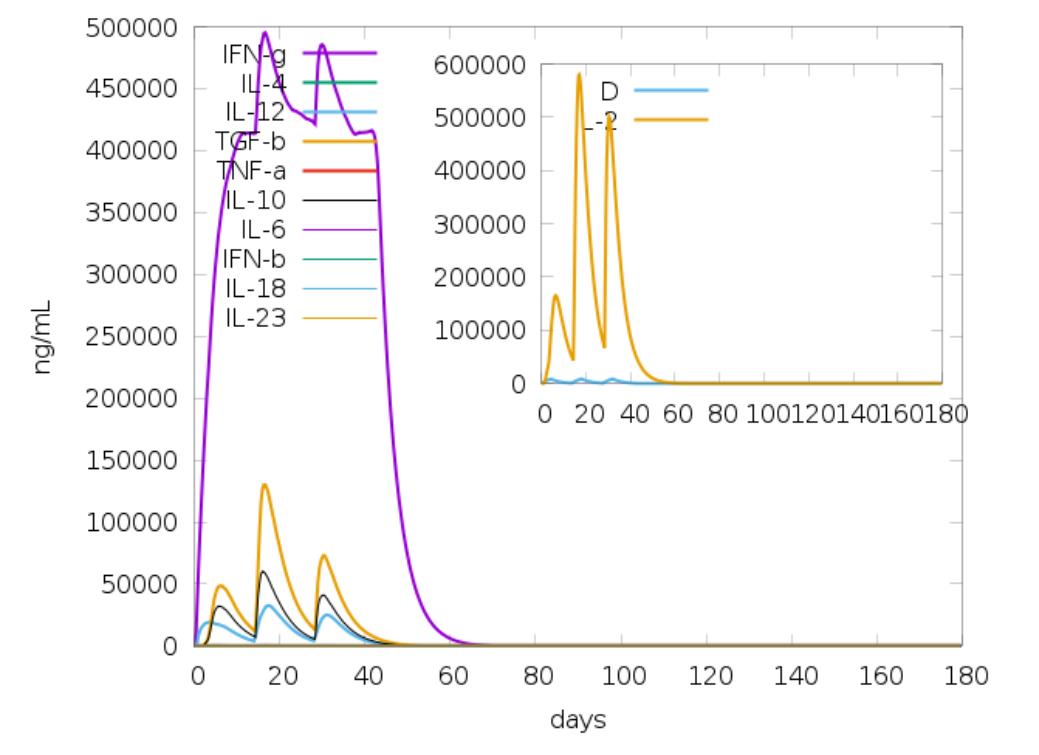


**Supplementary figure 2-5.** Concentration of cytokines and interleukins analyzed by C-ImmSim online server**.** XP_003392714.1 protein without any adjuvant was injected for three times at intervals of 2 weeks. IFN-gamma (IFN-γ), TGF-b (TGF-β), IL-10, and IL-12 are shown in purple line, yellow thick line, black line, and blue thick line respectively. IL-2 and danger signal (D) are presented in yellow thick line and blue thick line in the insert plot respectively.

**XP_003859056.1**


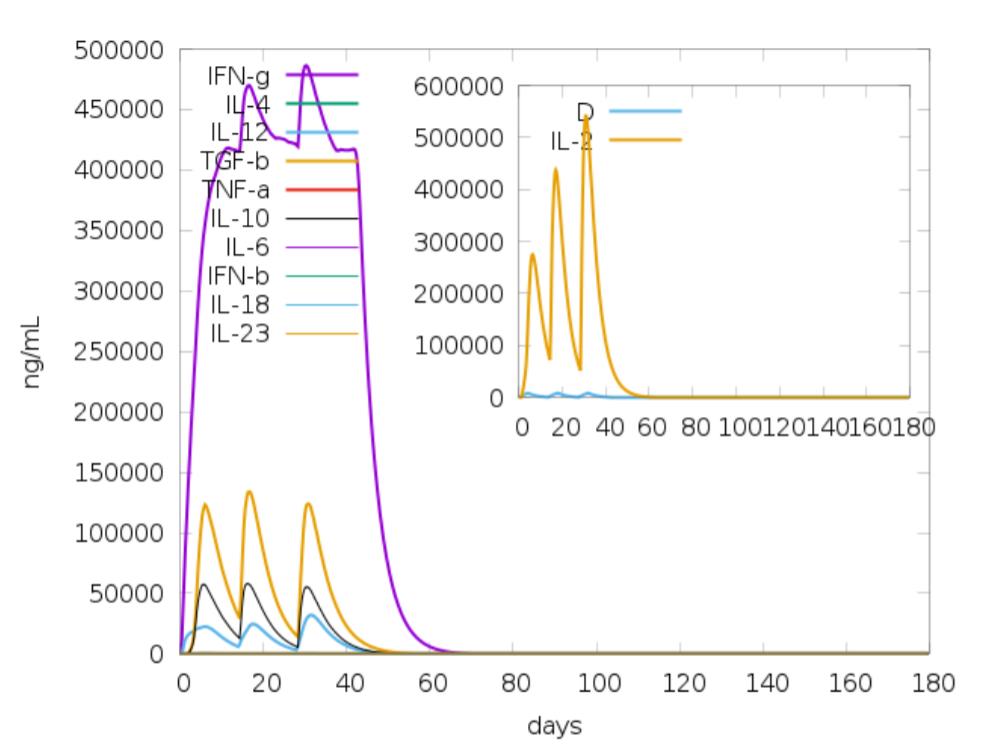


**Supplementary figure 2-6.** Concentration of cytokines and interleukins analyzed by C-ImmSim online server**.** XP_003859056.1 protein without any adjuvant was injected for three times at intervals of 2 weeks. IFN-gamma (IFN-γ), TGF-b (TGF-β), IL-10, and IL-12 are shown in purple line, yellow thick line, black line, and blue thick line respectively. IL-2 and danger signal (D) are presented in yellow thick line and blue thick line in the insert plot respectively.

**XP_003861362.1**


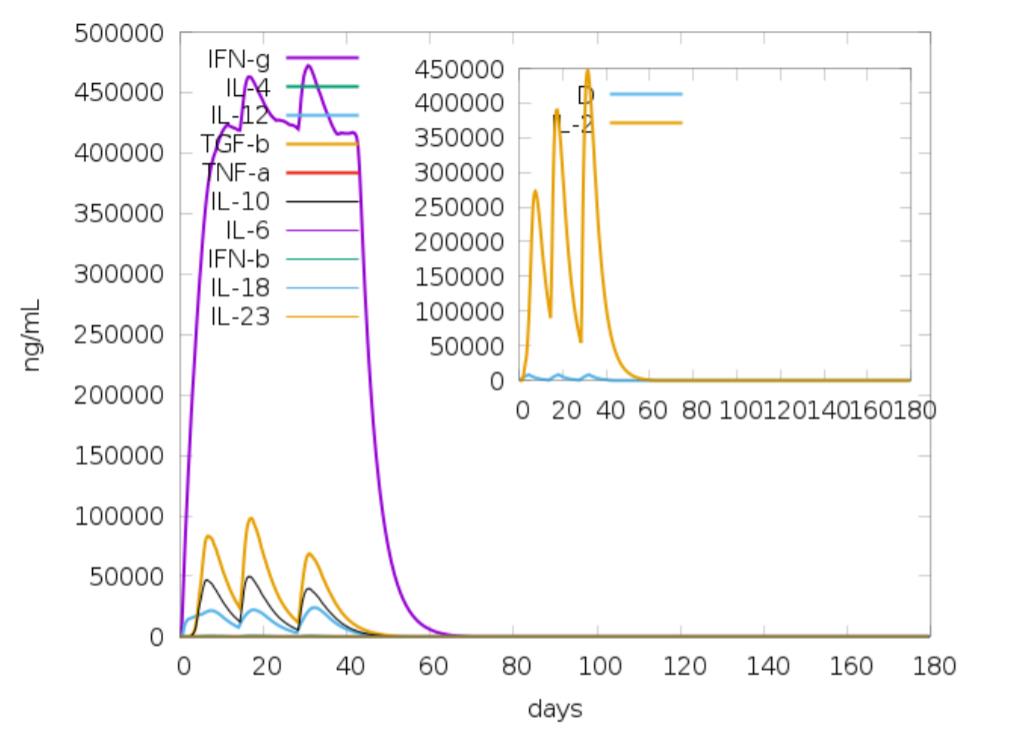


**Supplementary figure 2-7.** Concentration of cytokines and interleukins analyzed by C-ImmSim online server**.** XP_003861362.1 protein without any adjuvant was injected for three times at intervals of 2 weeks. IFN-gamma (IFN-γ), TGF-b (TGF-β), IL-10, and IL-12 are shown in purple line, yellow thick line, black line, and blue thick line respectively. IL-2 and danger signal (D) are presented in yellow thick line and blue thick line in the insert plot respectively.

**XP_003861510.1**


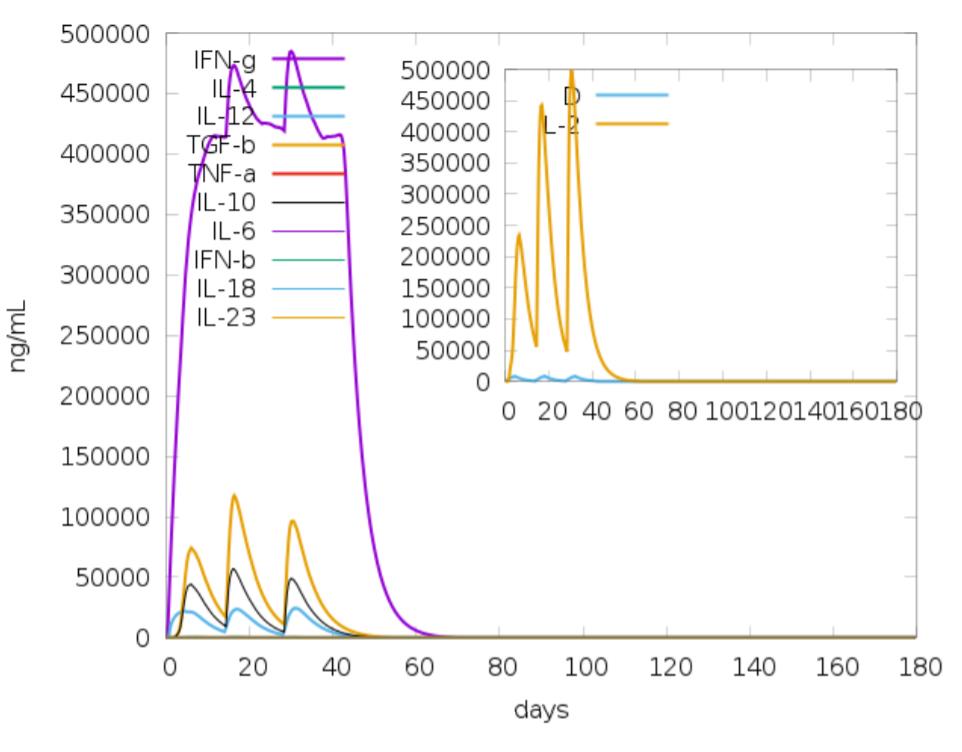


**Supplementary figure 2-8.** Concentration of cytokines and interleukins analyzed by C-ImmSim online server**.** XP_003861510.1 protein without any adjuvant was injected for three times at intervals of 2 weeks. IFN-gamma (IFN-γ), TGF-b (TGF-β), IL-10, and IL-12 are shown in purple line, yellow thick line, black line, and blue thick line respectively. IL-2 and danger signal (D) are presented in yellow thick line and blue thick line in the insert plot respectively.

**XP_003862806.1**


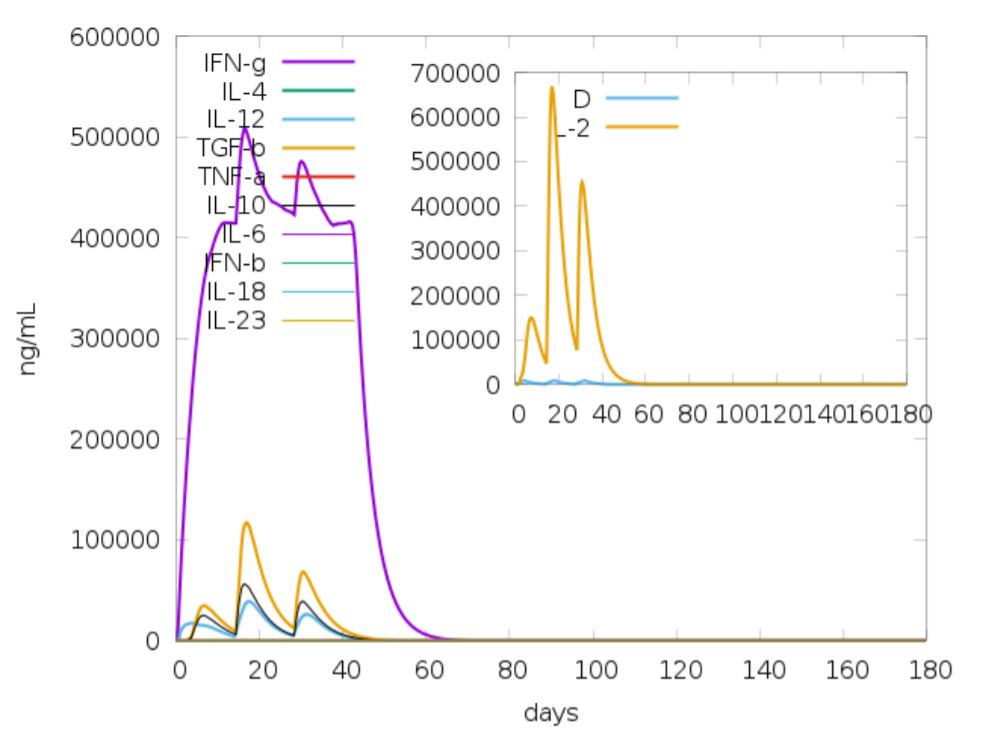


**Supplementary figure 2-9.** Concentration of cytokines and interleukins analyzed by C-ImmSim online server**.** XP_003862806.1 protein without any adjuvant was injected for three times at intervals of 2 weeks. IFN-gamma (IFN-γ), TGF-b (TGF-β), IL-10, and IL-12 are shown in purple line, yellow thick line, black line, and blue thick line respectively. IL-2 and danger signal (D) are presented in yellow thick line and blue thick line in the insert plot respectively.

**XP_003865405.1**


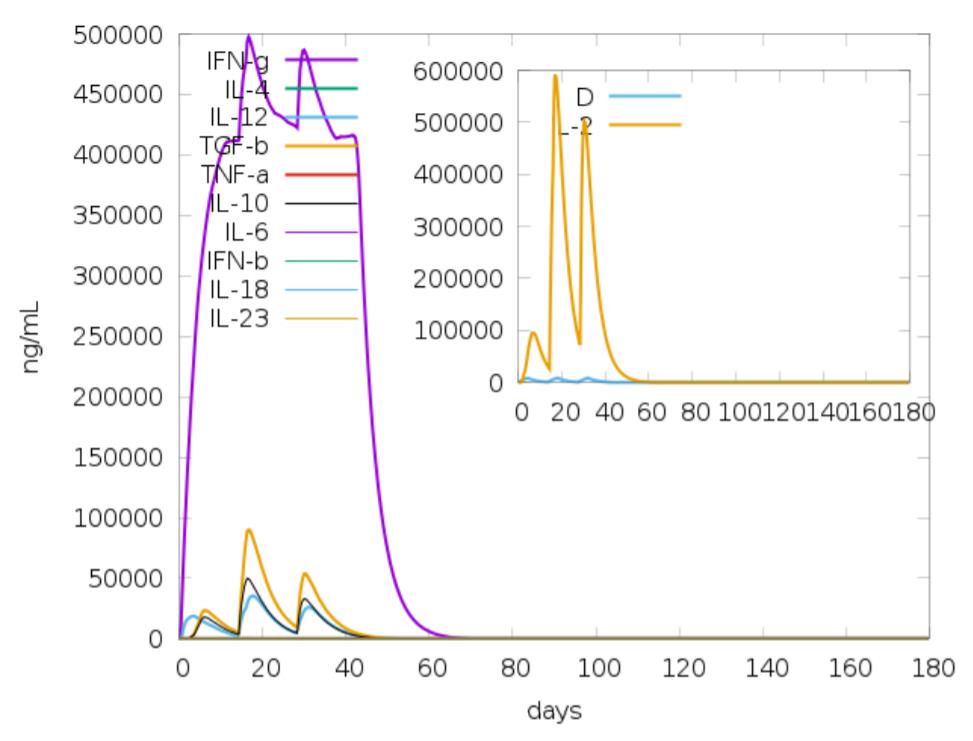


**Supplementary figure 2-10.** Concentration of cytokines and interleukins analyzed by C-ImmSim online server**.** XP_003865405.1 protein without any adjuvant was injected for three times at intervals of 2 weeks. IFN-gamma (IFN-γ), TGF-b (TGF-β), IL-10, and IL-12 are shown in purple line, yellow thick line, black line, and blue thick line respectively. IL-2 and danger signal (D) are presented in yellow thick line and blue thick line in the insert plot respectively.

**XP_003865225.1**


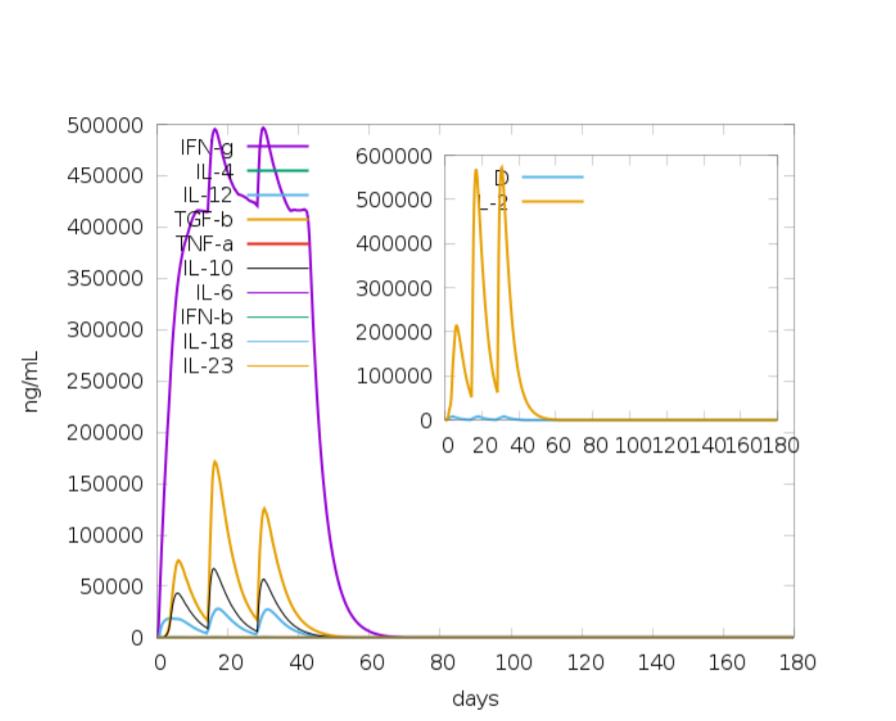


**Supplementary figure 2-11.** Concentration of cytokines and interleukins analyzed by C-ImmSim online server**.** XP_003865225.1 protein without any adjuvant was injected for three times at intervals of 2 weeks. IFN-gamma (IFN-γ), TGF-b (TGF-β), IL-10, and IL-12 are shown in purple line, yellow thick line, black line, and blue thick line respectively. IL-2 and danger signal (D) are presented in yellow thick line and blue thick line in the insert plot respectively.

**XP_003861228.1**


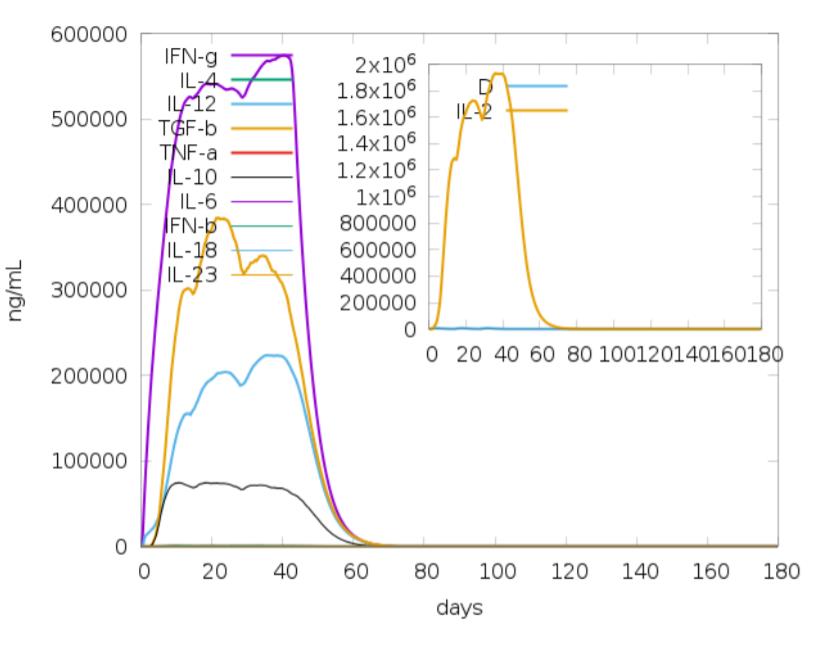


**Supplementary figure 2-12.** Concentration of cytokines and interleukins analyzed by C-ImmSim online server**.** XP_003861228.1 protein without any adjuvant was injected for three times at intervals of 2 weeks. IFN-gamma (IFN-γ), TGF-b (TGF-β), IL-10, and IL-12 are shown in purple line, yellow thick line, black line, and blue thick line respectively. IL-2 and danger signal (D) are presented in yellow thick line and blue thick line in the insert plot respectively.

**XP_003859515.1**


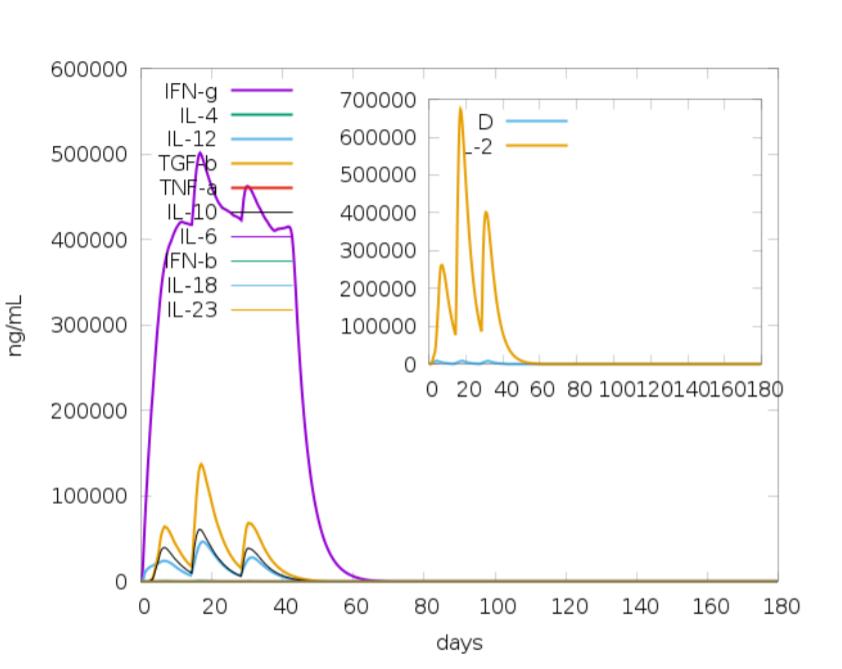


**Supplementary figure 2-13.** Concentration of cytokines and interleukins analyzed by C-ImmSim online server**.** XP_003859515.1 protein without any adjuvant was injected for three times at intervals of 2 weeks. IFN-gamma (IFN-γ), TGF-b (TGF-β), IL-10, and IL-12 are shown in purple line, yellow thick line, black line, and blue thick line respectively. IL-2 and danger signal (D) are presented in yellow thick line and blue thick line in the insert plot respectively.

**XP_001466815.1**


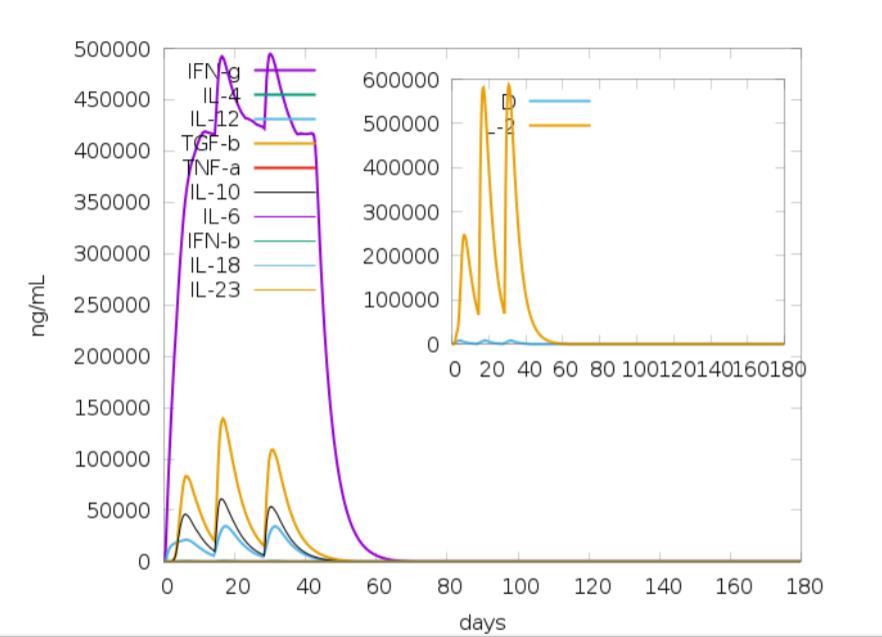


**Supplementary figure 2-14.** Concentration of cytokines and interleukins analyzed by C-ImmSim online server**.** XP_001466815.1 protein without any adjuvant was injected for three times at intervals of 2 weeks. IFN-gamma (IFN-γ), TGF-b (TGF-β), IL-10, and IL-12 are shown in purple line, yellow thick line, black line, and blue thick line respectively. IL-2 and danger signal (D) are presented in yellow thick line and blue thick line in the insert plot respectively.

**XP_003860857.1**


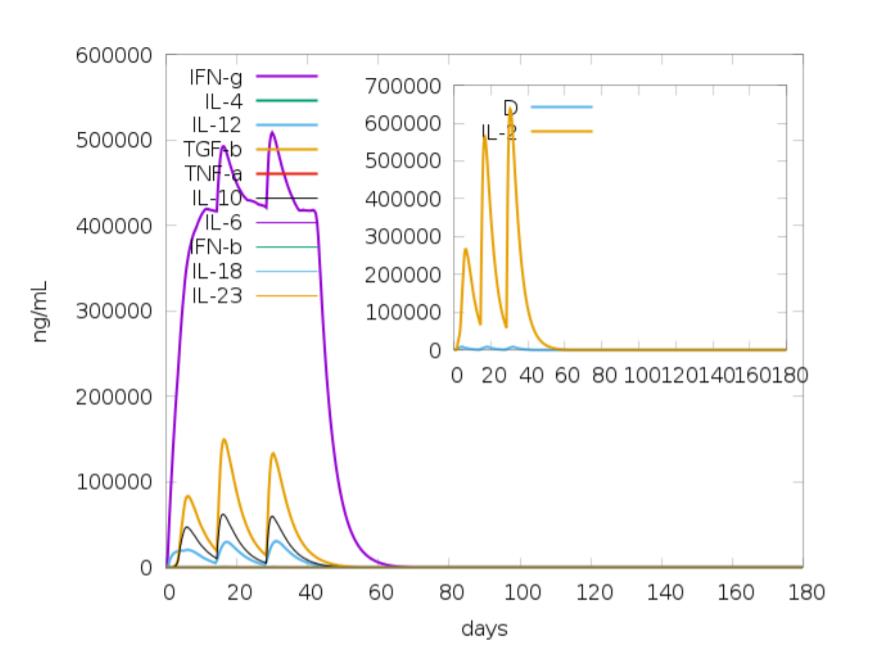


**Supplementary figure 2-15.** Concentration of cytokines and interleukins analyzed by C-ImmSim online server**.** XP_003860857.1 protein without any adjuvant was injected for three times at intervals of 2 weeks. IFN-gamma (IFN-γ), TGF-b (TGF-β), IL-10, and IL-12 are shown in purple line, yellow thick line, black line, and blue thick line respectively. IL-2 and danger signal (D) are presented in yellow thick line and blue thick line in the insert plot respectively.

**XP_003862657.1**


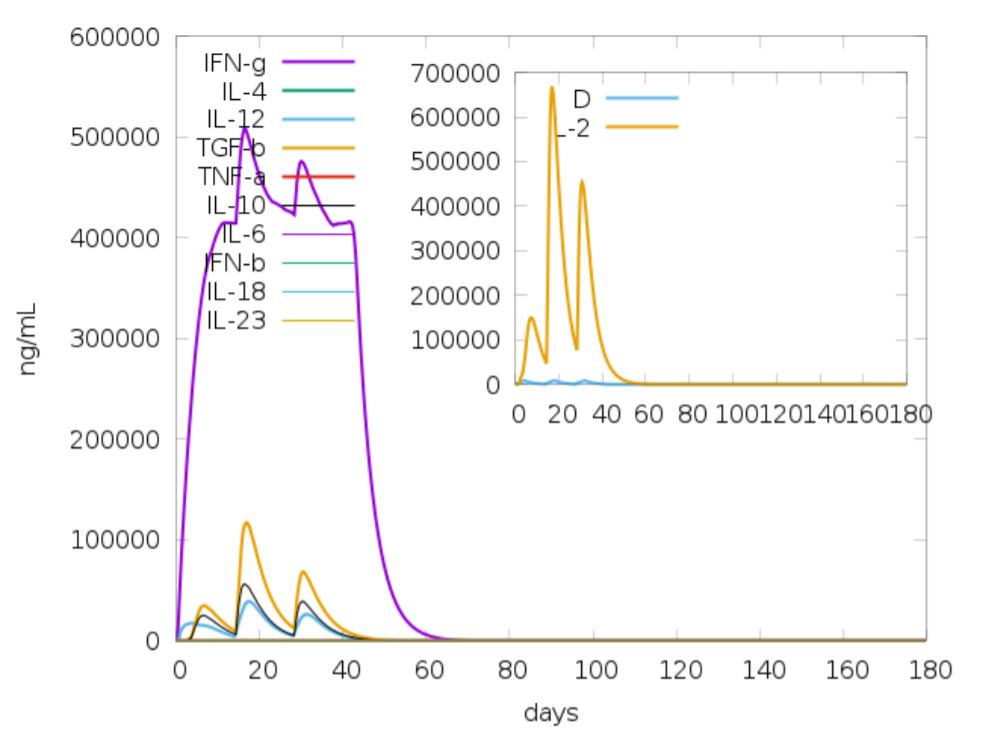


**Supplementary figure 2-16.** Concentration of cytokines and interleukins analyzed by C-ImmSim online server**.** XP_003862657.1 protein without any adjuvant was injected for three times at intervals of 2 weeks. IFN-gamma (IFN-γ), TGF-b (TGF-β), IL-10, and IL-12 are shown in purple line, yellow thick line, black line, and blue thick line respectively. IL-2 and danger signal (D) are presented in yellow thick line and blue thick line in the insert plot respectively.

**LeIF**


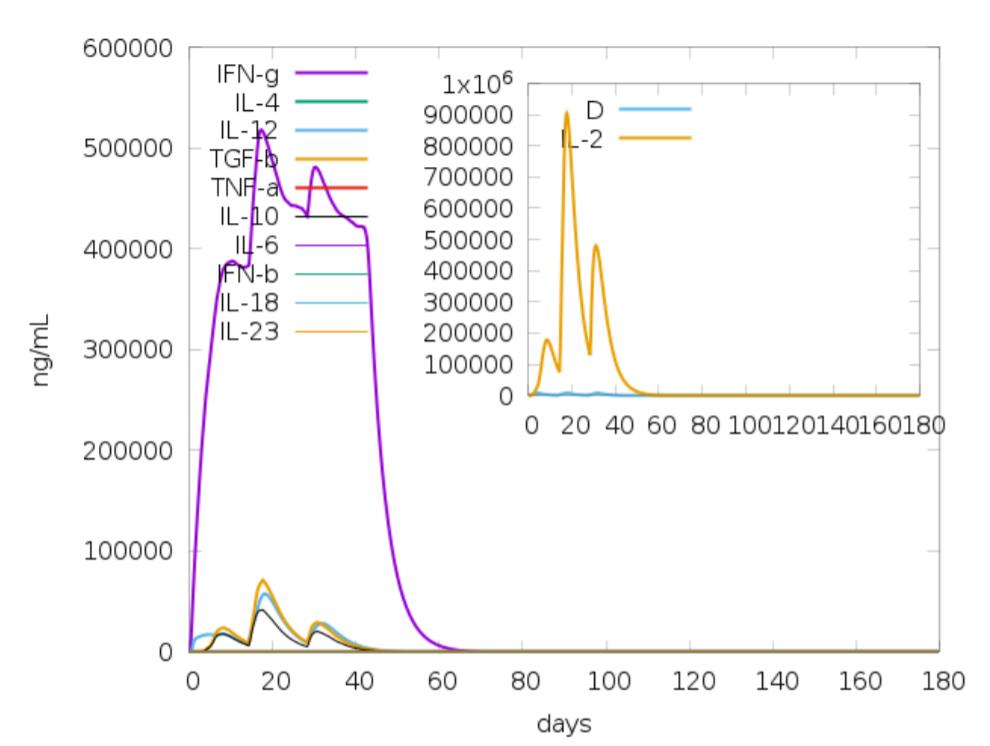


**Supplementary figure 2-17.** Concentration of cytokines and interleukins analyzed by C-ImmSim online server**.** LeIF protein without any adjuvant was injected for three times at intervals of 2 weeks. IFN-gamma (IFN-γ), TGF-b (TGF-β), IL-10, and IL-12 are shown in purple line, yellow thick line, black line, and blue thick line respectively. IL-2 and danger signal (D) are presented in yellow thick line and blue thick line in the insert plot respectively.

**Leish-111f**


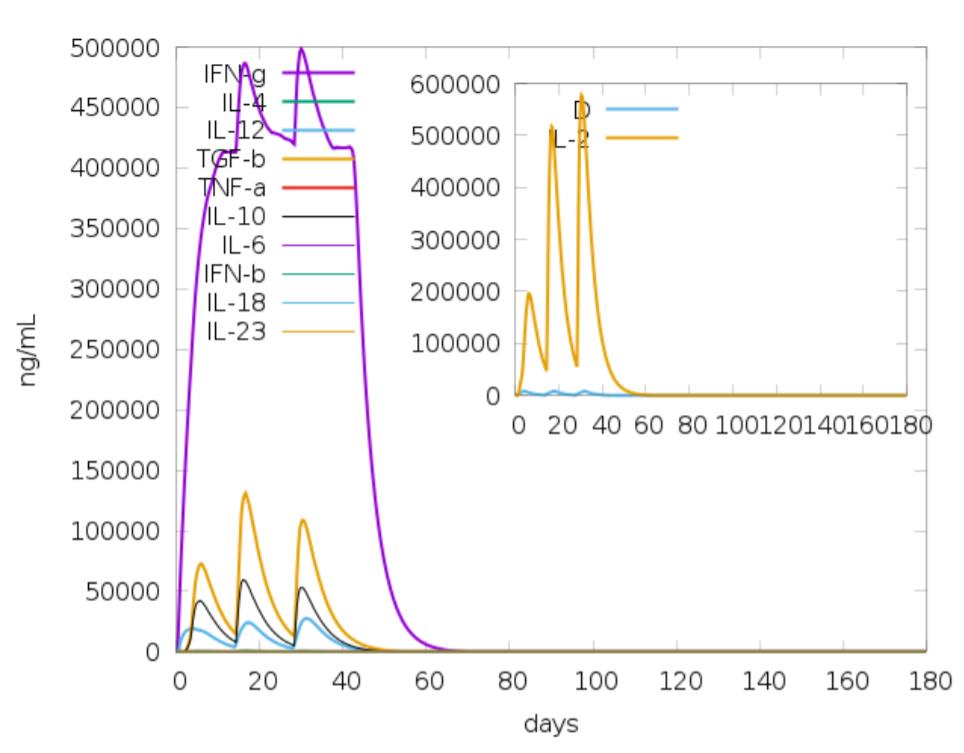


**Supplementary figure 2-18.** Concentration of cytokines and interleukins analyzed by C-ImmSim online server**.** Leish-111f protein without any adjuvant was injected for three times at intervals of 2 weeks. IFN-gamma (IFN-γ), TGF-b (TGF-β), IL-10, and IL-12 are shown in purple line, yellow thick line, black line, and blue thick line respectively. IL-2 and danger signal (D) are presented in yellow thick line and blue thick line in the insert plot respectively.

**LmST1**


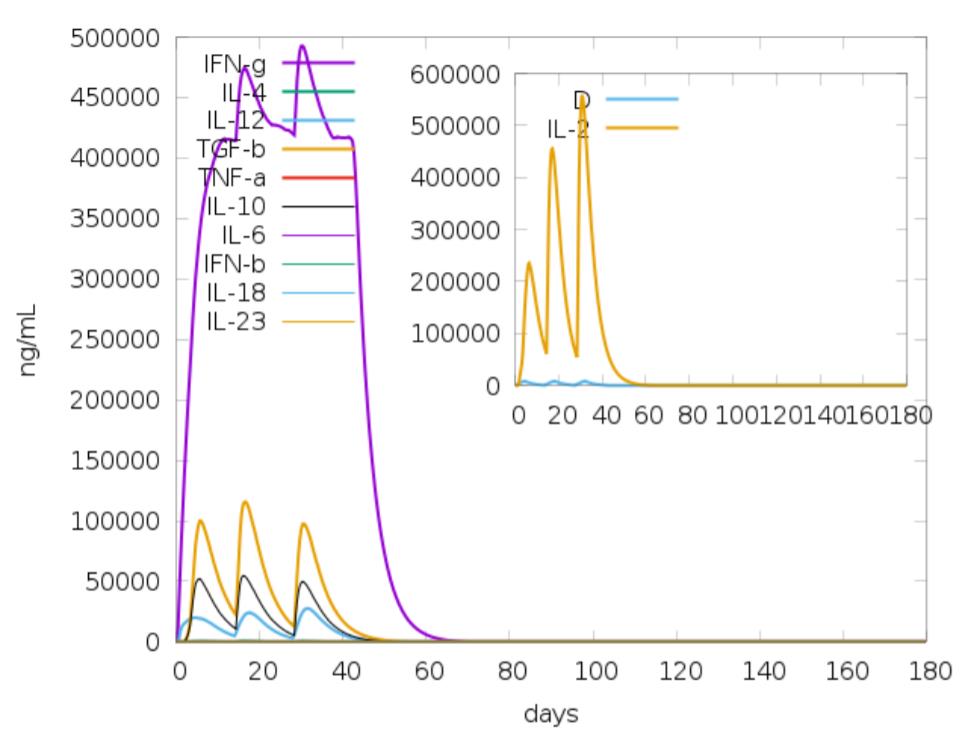


**Supplementary figure 2-19.** Concentration of cytokines and interleukins analyzed by C-ImmSim online server**.** LmST1 protein without any adjuvant was injected for three times at intervals of 2 weeks. IFN-gamma (IFN-γ), TGF-b (TGF-β), IL-10, and IL-12 are shown in purple line, yellow thick line, black line, and blue thick line respectively. IL-2 and danger signal (D) are presented in yellow thick line and blue thick line in the insert plot respectively.

**TSA**


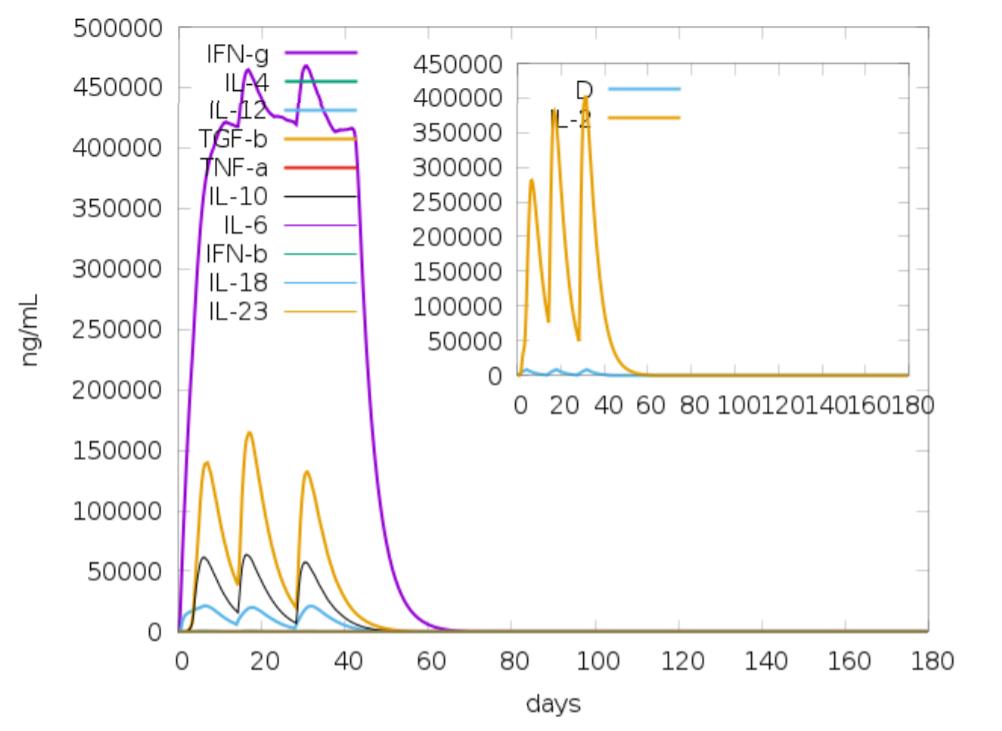


**Supplementary figure 2-20.** Concentration of cytokines and interleukins analyzed by C-ImmSim online server**.** TSA protein without any adjuvant was injected for three times at intervals of 2 weeks. IFN-gamma (IFN-γ), TGF-b (TGF-β), IL-10, and IL-12 are shown in purple line, yellow thick line, black line, and blue thick line respectively. IL-2 and danger signal (D) are presented in yellow thick line and blue thick line in the insert plot respectively.
